# Stapled BH3 Peptides against MCL-1: Mechanism and Design Using Atomistic Simulations

**DOI:** 10.1371/journal.pone.0043985

**Published:** 2012-08-31

**Authors:** Thomas L. Joseph, David P. Lane, Chandra S. Verma

**Affiliations:** 1 Bioinformatics Institute, A*STAR (Agency for Science, Technology and Research), Biopolis, Singapore, Singapore; 2 p53 Laboratory, A*STAR (Agency for Science, Technology and Research), Biopolis, Singapore; 3 Department of Biological Sciences, National University of Singapore, Singapore, Singapore; 4 School of Biological Sciences, Nanyang Technological University, Singapore, Singapore; Indian Institute of Science, India

## Abstract

Atomistic simulations of a set of stapled alpha helical peptides derived from the BH3 helix of MCL-1 (Stewart et al. (2010) Nat Chem Biol 6: 595–601) complexed to a fragment (residues 172–320) of MCL-1 revealed that the highest affinity is achieved when the staples engage the surface of MCL-1 as has also been demonstrated for p53-MDM2 (Joseph et al. (2010) Cell Cycle 9: 4560–4568; Baek et al. (2012) J Am Chem Soc 134: 103–106). Affinity is also modulated by the ability of the staples to pre-organize the peptides as helices. Molecular dynamics simulations of these stapled BH3 peptides were carried out followed by determination of the energies of interactions using MM/GBSA methods. These show that the location of the staple is a key determinant of a good binding stapled peptide from a bad binder. The good binder derives binding affinity from interactions between the hydrophobic staple and a hydrophobic patch on MCL-1. The position of the staple was varied, guiding the design of new stapled peptides with higher affinities.

## Introduction

Apoptosis is a conserved process that leads to cell death. Dysregulation of apoptosis contributes to disorders such as malignancies [1]. There are two recognized pathways that lead to apoptosis: “extrinsic” and “intrinsic” [2]. In both, a family of Cysteine Proteases, named Caspases act in a proteolytic cascade. The extrinsic pathway is controlled by extracellular events [3] while the intrinsic pathway begins when a cell is damaged beyond repair. The most characterized intrinsic pathway is mitochondrial and is controlled by the B-cell lymphoma 2 (Bcl-2) protein family [4]. The Bcl-2 protein family comprises suppressors (e.g., Bcl-2, B-cell lymphoma-extra large, or Bcl-XL myeloid cell leukemia sequence 1 or MCL-1) or promoters (e.g., Bcl2 associated X protein or Bax, Bcl-2 homologous antagonist/killer or Bak, BH3-only proteins including Bim, Bid) of apoptosis [5]. Various apoptotic stimuli trigger the release of factors (eg Cytochrome c) from the mitochondria that activate caspases. Bcl-2 related proteins appear to modulate the release of Cytochrome c [6].

MCL-1 is an anti-apoptotic member of the Bcl-2 family protein [7] and has been shown to be expressed in different cell types [8]. It promotes cell survival by inhibiting the apopototic cascade and is also found to be over-expressed in a variety of human cancers (B-cell lymphoma, chronic lymphocytic leukemia, chronic myeloid leukemia, etc) [9]. Further, tumors with high levels of anti-apoptotic members of Bcl-2 such as MCL-1 are often found to be resistant to chemotherapy [10]. Thus, inhibition of the function of the anti-apoptotic members of Bcl-2 such as MCL-1 may offer a novel avenue for designing anticancer drugs [11], [12].

**Figure 1:**
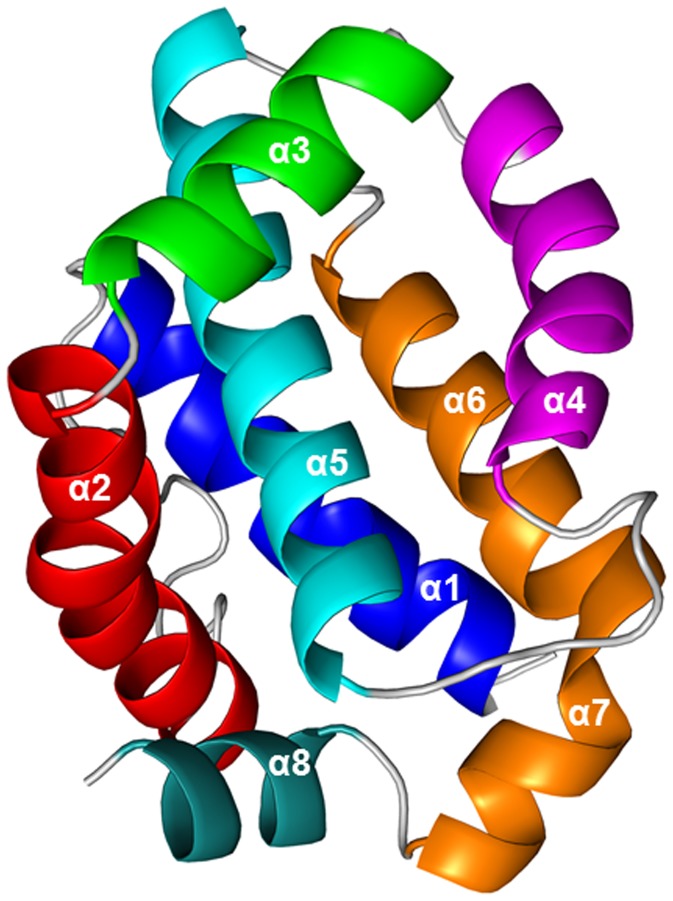
Ribbon diagram of unliganded MCL-1 showing the hydrophobic cleft formed by helices α2, α4, and α5.

The MCL-1 protein is 350 amino acids long and is homologous to BH (Bcl-2 homology) domains of the Bcl-2 family [7]. These domains are short motifs which mediate interactions between Bcl-2 proteins in modulating apoptosis [5]. MCL-1 has a BH3-binding groove (Figure 1) that is made up of portions of helices α3, α4, α5 (BH1), α8 (BH2) and α2 (BH3). In addition, there is a C-terminal transmembrane (TM) domain that localizes MCL-1 to the outer mitochondrial membrane [13] which is thought to be part of the apoptotic cascade; MCL-1 is also thought to localize to other intracellular membranes [14], [15], [16].

**Figure 2:**
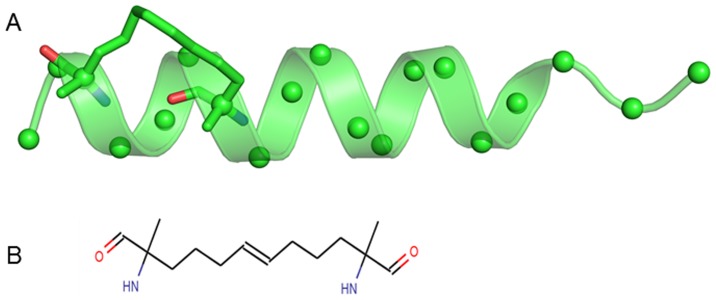
Structure of the staple. (A) The structure of the stapled BH3 peptide (BH3D) taken from its complex with MCL-1 as crystallized in the x-ray structure (3MK8) is shown in cartoon. The staple linking amino acid positions i and i+4 is shown in sticks and the C-α atoms are shown in spheres for clarity, (B) The chemical structure of the i, i+4 staple used.

As part of the strategy to inhibit these anti-apoptotic proteins, Abbott developed a small molecule (ABT-737) which targets Bcl-2 and Bcl-XL with high affinity but does not target MCL-1 [17], [18]. While this molecule has entered clinical trials, there are several small molecules [19], [20], [21], [22], peptides [23], and stabilized alpha helical peptidomimetics [24], that inhibit MCL-1 but are still in the investigational phases. A novel strategy to gain high affinity peptides has been developed by Verdine & coworkers and demonstrated its effectiveness initially for the BH3 system (Figure 2 A and B) [25]. This involved stabilizing a helical peptide with an appropriately placed hydrocarbon linker which was shown to preorganize the peptides into helices, stabilize the peptides against proteolytic degradation and make them cell permeable. In addition, computational models showed that the hydrocarbon staples can gain binding energy by interacting with hydrophobic patches on the surface of the target [26], [27]. To develop such inhibitors of MCL-1, Walensky and group identified a set of such peptides that inhibited MCL-1 both in vitro and in vivo [25], [28]. Structural characterization of the highest affinity peptide complexed to MCL1- showed that indeed the staple interacted with a hydrophobic part of the surface [29], [30], [31]. The technique of stapling peptides has now been shown to be effective in the p53 pathway [32], NOTCH pathway [33], BCL pathway [25], estrogen activation [34], cholesterol efflux [35], and in targeting HIV [36]. In addition, a successful strategy employing a double staple provides hope that this technique can also be used to recruit longer peptides [37].

As we had earlier successfully predicted using molecular dynamics (MD) simulations that the gain in affinity of the p53 peptides against MDM2 partly originated in interactions that the hydrocarbon staples make with hydrophobic patches on MDM2 [26] (later validated in a crystallographic study [38]), we decided to extend our studies to the report by Walensky and colleagues, where the position of the staple along a peptide against MCL-1 was varied [24]. MD simulations show that the interaction surfaces can be extremely dynamic [39], [40], [41] and hence help guide the careful placement of the staple in order to maximize affinity [26], [41] during the design of new peptides.

## Materials and Methods

The initial structure of MCL-1 bound to a stapled peptide was taken from the crystal structure 3MK8, resolved at 2.3Å [24]. The missing residues (K194-R201) were modeled using Modeler 9.7 [42] and guided by their positions in the NMR structure of MCL-1 bound to a peptide (PDB code 2KBW [43]). The starting model included residues 172–320 of human MCL-1, and residues 5–23 of the BH3 peptide [24]. The stapled regions were modeled using the Xleap module of AMBER [44] and the parameters were built using the antechamber module of AMBER [45], [46]. Only the N- and C- termini of MCL-1 were capped (with acetyl or ACE and N-methyl or NME respectively) to keep them neutral, in accord with the experiments [24]. Molecular dynamics simulations were performed with the SANDER module of the AMBER9 [44] package employing the all-atom ff99SB force field [47]. Simulations were carried out for the complexes of BH3 wild type and eleven stapled peptides bound to MCL-1 (Table 1). Each system was solvated with a TIP3P water box [48] whose sides are at a minimum distance of 10 Å to any protein atom. Particle Mesh Ewald method (PME) [49] was used for treating the long range electrostatics. All bonds involving hydrogen atoms were constrained by SHAKE [50]. A time step of 2fs was used. Initially, the whole system was minimized for 4,000 steps, to remove any unfavorable interactions. Subsequently, the systems were each heated to 300 K for 30 ps under NPT conditions. After this, each system was equilibrated for 100 ps and then simulated for 20 ns at constant temperature (300 K) and pressure (1 atm) and structures were stored every 1 ps. The free energy of binding (ΔG_bind_) of the peptides to MCL-1 was computed using the MM-GBSA (molecular mechanics/Generalized Born surface area) method [51], [52] using the GB module [53] in Amber while the non-polar component was estimated from the solvent accessible surface area using MOLSURF [54] with ΔG_solv,np_ = 0.00542*SASA +0.92 [55]. Each energy term was averaged over frames taken every 2 ps over the last 10 ns of each simulation. Vibrational entropy was estimated using normal mode analysis (Nmode module of Amber) [56] and averaged over 200 ps intervals. PyMOL [57] and Visual Molecular Dynamics [58] (VMD) were used for visualizations.

**Table 1:** The sequences of BH3 peptide and stapled BH3 peptide analogs taken from Stewart [24] used in this study.

Peptide	Sequence																		
BH3wt	A	L	E	T	L	R	R	V	G	D	G	V	Q	R	N	H	E	T	A
BH3A	A	L	E	T	L	R	St	V	G	D	St	V	Q	R	N	H	E	T	A
BH3B	A	L	St	T	L	R	St	V	G	D	G	V	Q	R	N	H	E	T	A
BH3C	A	L	E	T	L	R	R	V	St	D	G	V	St	R	N	H	E	T	A
BH3D	A	L	E	T	L	R	R	V	G	D	G	V	St	R	N	H	St	T	A
BH3E	A	L	E	T	L	R	R	V	G	D	G	V	Q	R	St	H	E	T	St
BH3F	A	L	E	T	L	R	R	V	G	D	G	V	St	R	N	H	St	D	A
BH3G	A	L	St	T	L	R	St	V	G	D	G	V	Q	St	N	H	E	St	A
BH3H	A	L	E	St	L	R	R	St	G	D	G	V	St	R	N	H	St	T	A
BH3I	D	L	E	St	L	R	R	St	G	D	G	V	St	R	N	H	St	T	A
BH3J	D	L	E	St	L	R	R	St	G	D	G	V	St	D	N	H	St	T	A
BH3K	A	L	E	St	L	R	R	St	G	D	G	V	Q	R	N	H	E	T	A

St-XXX-St refers to the hydrocarbon linker (as shown in Figure 2B).

## Results and Discussion

**Figure 3:**
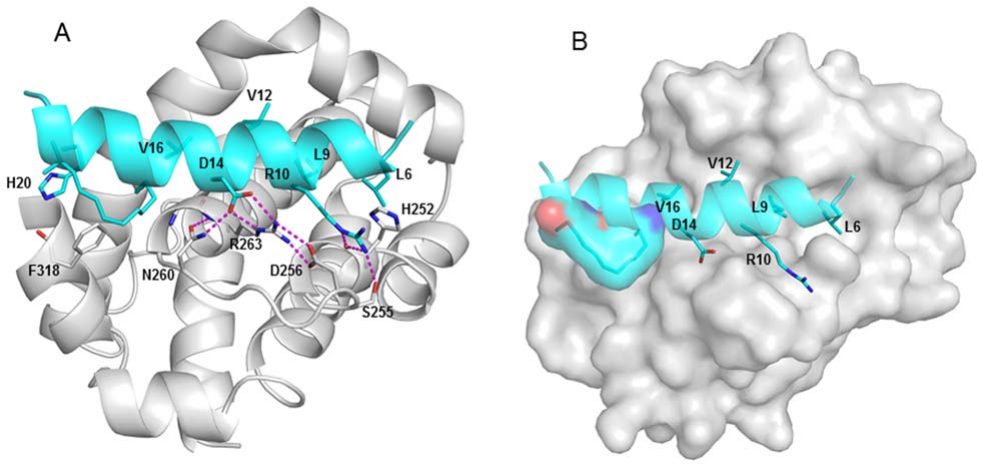
The structure of MCL-1 (shown in grey) bound to BH3D peptide taken from the crystal structure 3MK8 [24]. (A) The Arg10 sidechain is stabilized by the His252 backbone. The interaction of Asp14 with Arg263, and the hbond cluster comprising the sidechains Asp256, Asn260 and Arg263 with Asp14 are well maintained (shown in cartoon), (B) The packing of the BH3D staple against the hydrophobic residues of MCL-1 shown in surface.

We have carried out MD studies investigating the binding of 6 BH3 peptides to MCL-1; these peptides have been experimentally characterized by Walensky and colleagues [24]. The peptides include the wild type (wt) peptide and 5 stapled peptides which are labeled MCL-SAH-A to MCL-SAH-E respectively in Figure 2c that appears in the work reported by Walensky et al. [24]. Our simulations of the interactions of these peptides with MCL-1 guided the design of an additional six stapled BH3 peptides (which we shall refer to as BH3F-BH3K; we will further refer to the wild type peptide as BH3wt and their 5 stapled peptides as BH3A-BH3E (Table 1)). Walensky and coworkers initially designed a peptide (BH3A) that displayed 43 nM affinity against MCL-1. They subsequently subjected this to an alanine scan to determine the positions where staples could be introduced, while minimizing perturbations to the interactions with MCL-1. This yielded a set of 4 stapled peptides with affinities ranging from 10–33 nM with BH3D displaying the highest affinity for MCL-1. The complex of BH3D bound to MCL-1 was subsequently resolved using crystallography [24]. This structure revealed an interaction between the hydrophobic staple and a hydrophobic patch on MCL-1 that was hypothesized to be responsible for part of the enhanced affinity (Figures 3A and B); a similar feature was predicted for the p53 stapled peptides with high affinity against MDM2 that were characterized by the Verdine group [38] by our simulation studies [26]. Indeed, the prediction of our simulations found close agreement in the recently described crystal structure of MDM2 complexed to a stapled peptide [38], thus lending support to our simulation strategy.

In order to benchmark our calculations, we used the crystal structure of MCL-1 complexed to BH3D, mutated BH3D to generate BH3A, and then subjected BH3A to a computational alanine scan [59]. As expected, mutating residues that are buried (Figure S1 A and B) including L6A (L210A in the paper by Walensky & colleagues [24]; henceforth the number in parentheses will refer to this), L9A (L213A) and V16A (V220A) destabilize the binding energies by ∼3–5 kcal/mol. In contrast, the mutations R10A (R214A) and D14A (D218A) undergo much greater destabilization (∼12–20 kcal/mol) resulting from the loss of extensive hbond networks that they are part of (as can be seen in Figure S1A and Figure 3A). The computed affinities of the Ala mutants for MCL-1 show a trend that mirrors the experimental findings (Table S1) and establish an appropriate benchmark.

All the simulations were judged to be stable based on the time evolution of the root-mean-square deviation (RMSD) and is given as Figures S2, S3 and S4 and the radius of gyration of the protein and peptides, given as Figures S5, S6 and S7. The binding energetics show that the computed affinities of all except the poorest peptide are similar (Table 2 and Table S2). Our computations, in agreement with the experimental data, also reveal BH3C as the lowest affinity peptide. The inability of the simulations to accurately reproduce the trend in the experimental affinity is due to the small range of experimental affinities between BH3A and BH3D, which is 10 to 43 nM [24]. This translates into a free energy range of −11 to ∼−10 kcal/mol which is too small to be accurately captured by current computations. While efforts are ongoing to improve the computation of absolute binding affinities [60], nevertheless the current state of the technology is reliable only in as far as a match is obtained in the trends seen in experiments or in some computed parameter that matches the experimental trend. The quantitative accuracy of computations currently are limited by various factors including force field parameters, insufficient sampling, statistical errors, convergence, computations of entropies [61], [62], [63], [64], while some progress has been reported with longer simulations in terms of sampling [65] nevertheless, the simulation setup is still quite limited in its ability to mimic experimental conditions including changing pH, salt effects etc. Further uncertainties arise from differences in crystallographic structures, low resolutions, incompletely resolved structures, and lack of detailed thermodynamic decompositions of interactions including enthalpic and entropic contributions which could be determined using Isothermal Calorimetry combined with Surface Plasmon Resonance. Nevertheless, simulations are a powerful tool to yield structural insights that rationalize observed trends as has also been shown in several other systems [26], [32], [38] and are proving useful to guide new experiments [66].

**Table 2:** Binding free energy (in kcal/mol) of MCL-1 with wt and stapled BH3peptides.

Δ(binding)	BH3wt	BH3A	BH3B	BH3C	BH3D	BH3E	BH3F	BH3G	BH3H	BH3I	BH3J	BH3K
ΔH	−67.4(5.3)	−66.8(6.2)	−62.5(8.3)	−52.9(5.2)	−63.2(4.7)	−68.1(4.6)	−64.6(4.9)	−68.2(5.4)	−68.7(4.7)	−71.7(5.9)	−70.3(4.5)	−72.7(4.7)
−TΔS	40.1(5.6)	38.8(5.4)	36.9(5.3)	41.6(6.3)	36.9(4.6)	41.3(4.4)	38.4(5.4)	41.7(4.2)	35.4(5.1)	38.3(5.6)	38.5(4.5)	39.0(5.3)
ΔG_bind_	−27.3	−27.9	−25.6	−11.2	−26.3	−26.8	−26.1	−26.4	−33.4	−33.5	−31.7	−33.7

We first examine the complex of BH3D, the peptide with the highest affinity against MCL-1. This peptide was derived from the α2 helix (_208_KALETLRRVGDGVQRNHETAF_228_) of the BH3 domain of MCL-1. In the complexed state, it exists as a short amphipathic α-helix, engaging the BH3-binding groove of MCL-1 with additional contacts between the staple and a hydrophobic patch on MCL-1 (Figures 3A and B). Although variants of BH3 that are active against MCL-1 have been reported [24], [43], [67], [68], BH3D displays the highest affinity. The crystallographic data [24] shows that hydrophobic residues Leu6, Leu9, Val12 and Val16 of BH3D lie buried deep inside the hydrophobic groove of MCL-1. These interactions are further strengthened by several hbond networks. These include: sidechain of Asp14 in BH3D makes hbonds with sidechains of Arg263 and Asn260 of MCL-1, and Arg263 in turn engages in a salt bridge with Asp256 of MCL-1; sidechain of Arg10 of BH3D hbonds with Ser255 sidechain and His252 backbone of MCL-1; BH3D Glu7 makes an hbond with His252 of MCL-1 and, NE2 in His20 of BH3D hbonds with backbone of Phe318 of MCL-1.

Our simulations reveal similar hbonding patterns along with some differences. The Glu7-His252 interaction is replaced with His252 making a transient hbond with the backbone of Leu6 while the Glu7 sidechain prefers to be solvated with an occasional salt bridge with Arg11. The Arg10 sidechain is stabilized by the His252 backbone throughout the simulation. The interaction of Asp14 with Arg263, and the hbond cluster comprising the sidechains Asp256, Asn260 and Arg263 with Asp14 are stable throughout the 20ns. The interaction of His20 sidechain with the Phe318 backbone exists for 96% of the simulation time (Figures 3A and 3B).

It is clear that the peptide is well sequestered in the binding pocket and doesn’t undergo any large conformational rearrangements. The reproduction of the crystallographically observed characteristics of the interactions between the peptide and the receptor suggest that the simulations are well behaved.

### Peptides in Solution

All simulations were judged to be stable based on the time evolution of the RMSD (Figure S3) and radius of gyration (Figure S6). Root-mean square fluctuations (RMSF) (Figure S8) of all peptides remain similar and as expected, the regions constrained by the staples show lower fluctuations. It is interesting that the poor binding peptide BH3C shows higher helicity compared to the good binders like BH3A, BH3D and BH3E peptides (Figure S9A–L).

In the wt peptide simulations (Figure S10A), the helicity extends from Leu6–Gln17 (crystallographically observed) to Leu6–Glu21. This results from hbonds between the sidechains of Asp14 and Arg18. The positively charged Arg18 interacts with the negatively charged Asp14 and this is complemented by an hbond between Thr18 and Asp21. The backbone of Arg18 forms an hbond with the sidechain of Thr22, which makes this peptide more helical at its C-terminus. At the N-terminus, the Thr8 sidechain interacts with the backbone of Ala5 to make this region helical too. In BH3A, Arg11 is replaced with the staple, which makes this peptide more negatively charged and with somewhat reduced helicity (compared to BH3wt). The staple also removes stabilizing interactions of Glu7 with Arg11 and renders the region highly mobile; with the staple localized to the middle of the helix, the charged ends prefer to be solvated and hence are highly mobile. In BH3B, the staple is located at the N-terminus which makes this region more helical; the interactions of the charged residues Asp14, Arg18 and Glu21 at the C-terminus make this region more helical; the intervening region is not helical. BH3C (Figure S10B) peptide is the most helical among all the unbound peptides analyzed, and interestingly is also the only inactive peptide. In this peptide the staple is placed in the middle of the entire sequence. The increased helicity arises from enhanced helicity at the terminal regions promoted by stable interactions between the sidechains of Glu7 and Arg11 and between the sidechains of Arg18 and Glu21. The localization of the staple in the centre prevents Asp14 from interacting with Arg11 and Arg18. In BH3D (Figure S10C), Glu21 is replaced with the staple, which makes this peptide positively charged and also has somewhat reduced helicity (compared to BH3wt). The removal of Glu21 removes the stabilizing interactions of Arg18 and this in turn interacts with negatively charged Asp14. The staple also appears to lead to an hbond between the sidechain of Thr22 and the backbone of Arg18, further imparting helicity to the C-terminal region. While in the BH3E peptide, although the staple is located in the C-terminal region, the charged residues in the N-terminal region appear to induce helicity in this region resulting in helicity in both the N-terminal and C-terminal regions; the region in between remains unstructured or in a loop conformation. In summary, all peptides are equally helical; BH3C is most helical and yet least active.

### Simulations of the MCL-1-Peptide Complexes

#### Secondary structure of the peptide in the complex

In contrast to the peptides in solution, when complexed to MCL-1, all peptides except BH3C are helical (Figures S11A–L), especially in the Glu7–Thr22 region (throughout the 20 ns). So what is the reason for this paradoxical behavior?

We find that upon complexation, the staple in the poorest binder BH3C is located in a position which leads to maximal disruption of the hbond network that has been highlighted above. The introduction of the staple at Gly13 leads to a loss of the hbonds that are made between Asp14 of the peptide and Asn260/Arg263 of MCL-1; the sidechain of Asp14 interacts instead with the Arg18 sidechain in BH3C, leading to a strain that results in reduced helicity of BH3C in its C-terminal region. However, the His252 backbone-Arg10 sidechain, Ser255 sidechain-Arg10 sidechain, and His20 sidechain-Phe318 backbone interactions are conserved, albeit with a reduced lifetime. The other end of the staple replaces Gln17 and this leads to a loss of the hbond with Gly262. In general, all the peptides, except BH3C, show improved helicity in the bound state relative to their free states, as is evident from the temporal evolution of the secondary structures (Figures S11A–L); BH3B, BH3D and BH3H are most helical.

#### Key interactions in the MCL-1-Peptide complexes

Overall RMSF of all the complexes remain similar (Figures S12 and S13) the only real differences are seen in the peptides. As expected, the peptides show lower fluctuations either at the 3 amino acids, Leu6, Leu9 and Val26 that are deeply embedded in MCL-1 or in the regions that are constrained by the staples.

The WT simulation shows the 8–19 region as helical throughout the 20 ns, as also is the case in BH3B, BH3D, BH3F and BH3H. The other peptides show the following regions as helical: 11–21 (BH3A), 7–21 (BH3E), 7–19 (BH3G), 7–22 (BH3I), 10–21 (BH3J and BH3K). This appears to be in accord with Walensky et al. [24], who designed the peptides with a view to achieving higher affinity through enhanced helicity of the peptides, especially by the introduction of i, i+4 staple. Similar features characterized the design and affinities of peptides for the p53-MDM2 and estrogen receptor systems [26], [32], [38].

**Figure 4:**
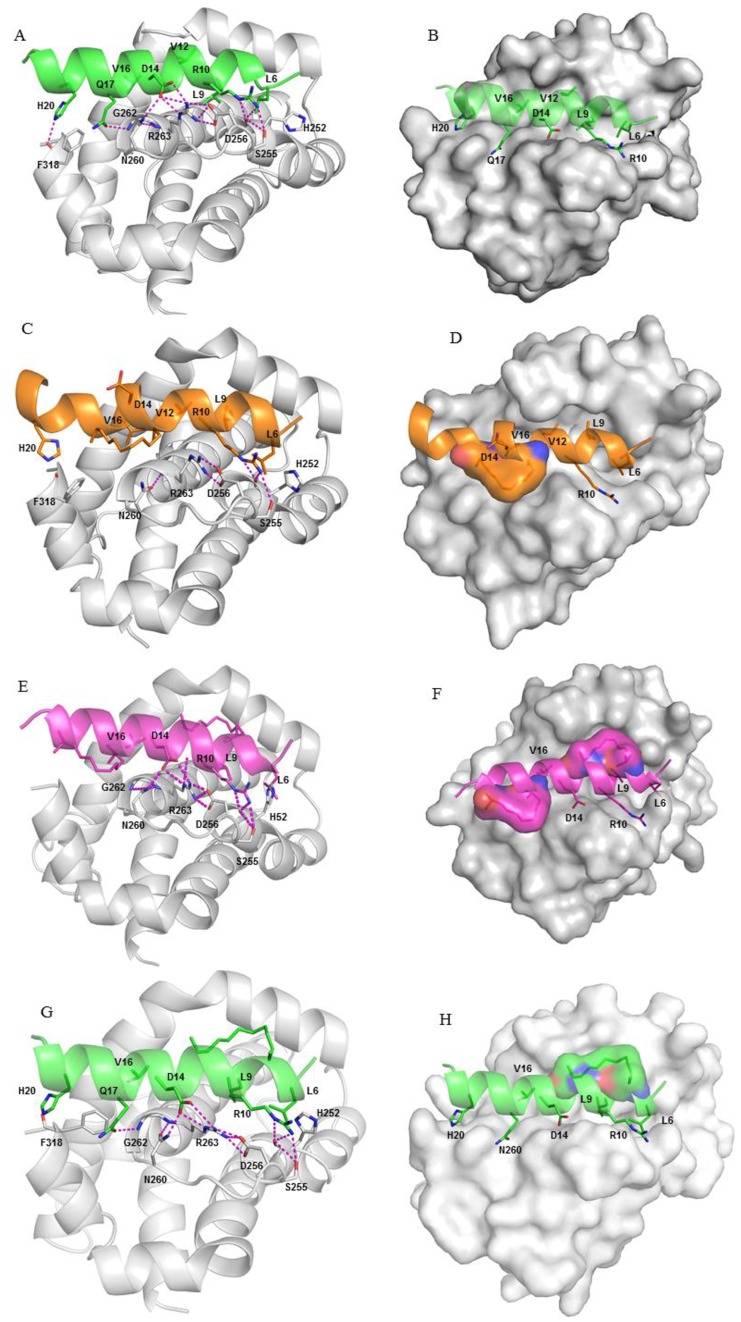
BH3wt bound to MCL-1 (shown in grey). (A) Asp14 of the peptide interacts with Arg263 and Asn260 of MCL-1; Arg263 also interacts with Asp256; Arg10 hbonds with Ser255; His20 sidechain hbonds with the backbone of Phe318; Gln17 sidechain hbonds with the backbone of Gly262 (B) The hydrophobic groups Leu6, Leu9, and Val16 are buried in the hydrophobic binding groove on the surface of MCL-1 (shown in surface); BHC bound to MCL-1 (shown in grey) (C) The location of the staple forces it to point into the MCL-1 surface creating a steric clash, thus creating a strain on the backbone of the BH3C peptide and its helicity. The loss of key hbond networks result in decreased contributions from Arg10 and Asp14 when compared with BH3wt peptide (shown in cartoon), (D) MCL-1 bound to BH3C peptide (shown in surface); BH3H bound to MCL-1 (shown in grey) (E) Double stapling improves the packing of the stapled regions and also maintains the helical content (in cartoon) (F) The hydrophobic groups Leu6, Leu9, and Val16 are buried in the hydrophobic binding groove on the surface of MCL-1 (shown in surface); BH3K bound to MCL-1 (shown in grey) (G) This staple interacts with the hydrophobic patch on the MCL-1 surface but also enables Gln17 to stabilize the system by hbonding to the backbone of Gly262 (shown in cartoon) (H) The hydrophobic groups Leu6, Leu9, and Val16 are buried in the hydrophobic binding groove on the surface of MCL-1 (shown in surface).

In the MCL-1 - BH3wt complex (Figures 4A, 4B) the interactions that engage Arg10 and Asp14 and Leu6, Leu9 and Val16 in BH3D (Figures 3A and B) are maintained. There are additional interactions (legend to Figures 4A and B) and (Movies S1 and S2). The charged residues Arg11, Arg18 and Glu21 prefer to be solvated. Significant contributions to the binding energy are made by key hydrophobic residues Leu6, Leu9, Val12 and Val16 (−5.5, −4.9, −3.2 and −2.9 kcal/mol respectively), and by polar residues Arg10, Asp14 and Gln17(−5.4, −3.1 and −1.7 respectively), (Table 2; Tables S2 and S3).

From the simulations of the complexes, visual inspection immediately shows that the good binders and the poor binder can be separated based on the location of the staples as this appears to determine their orientations and the associated interactions in the complexes.

In the non-binder (BH3C), the staple is located in order to avoid clashes with the surface of MCL-1, but the peptide is distorted from helicity. In contrast, the good binders have the peptides in helical conformations and their staples either “draped” over the surface of MCL-1, or in close proximity and clearly enhance the affinity of the peptides by these additional interactions (Table 2). There appear to be two major drivers of the high affinities: (a) gain in interaction energy of the peptide as a result of the MCL-1-staple interaction; (b) decrease in the penalty paid for hydrating the hydrophobic staple. In addition, there is the reduced entropic penalty for immobilizing the peptides onto the surface of MCL-1 by the staple-induced pre-organization into helical motifs as we have seen in the section on peptide simulations.

BH3 peptides [24], [43], [67] derived from BID, BIM and NOXA have also been shown to bind to MCL-1, and have hydrophobic residues at homologous positions. Residues I86/I148, L90/L152/L78, V93/I155/I81 and M97/F159/V85 are buried deeply inside the BH3 binding groove of MCL-1; in Walensky's BH3 peptides, Leu6, Leu9, and Val16 are buried while Val12 is partially exposed.

In BH3wt, Arg11 and Gly15 make no contribution to the binding since Arg11 is well solvated. Hence replacing these residues to form the staple of BH3A would in principle be tolerated (Figures S1A and B). Simulations show that helicity of BH3A decreases in its N-terminal region (Figure S11B). Arg10 and Asp14 maintain the hbond cluster as seen for BH3D. The hydrocarbon staple is solvent exposed, but it contributes to the binding significantly (−1.9 kcal/mol); in contrast Arg11 and Gly15 contribute negligibly in BH3wt. In BH3A, significant contributions were made by key hydrophobic residues Leu6, Leu9, Val12 and Val16 (−5.5, −5.0, −3.0 and −3.1 (kcal/mol) respectively), and also by polar residues Arg10, Asp14 and Gln17 (−5.3, −3.4 and −2.2 (kcal/mol) respectively), to the binding energy. Overall the computed binding affinity of BH3A is similar in strength to that of BH3wt (Table 2; Tables S2 and S3).

In BH3B, the staple replaces Glu7 and Arg11, which results in better helicity. With the staple pointing into solvent, the interactions remain similar to those of BH3A (Figures S14A and B). The Glu7 sidechain makes transient interactions with the N- terminal in BH3wt, which is lost upon the introduction of the staple at position 7. This constrains the Nterminal region into a helical state (Figure S11C), leading to reduced mobility. The presence of the staple reduces the interactions of Arg10 with His252 and Ser255 (the lifetimes are reduced from 89% to 75% of the simulation time) Asp14 maintains the network seen for BH3D whilst Gln17 makes an hbond interaction with Gly262.

The staple contributes 1 kcal/mol more than the staple in BH3A. However Glu7 contributes ∼1.6 kcal/mol in BH3A and so the net result of replacing Glu7 by the staple in BH3B is actually destabilizing compared to BH3A. In the BH3B peptide, significant contributions were made by key hydrophobic residues i.e., Leu6, Leu9, Val12 and Val16 (−5.7, −5.1, −2.9 and −3.2 (kcal/mol) respectively), and also by polar residues i.e., Arg10, Asp14 and Gln17 (−4.3, −2.9 and −2.2 (kcal/mol) respectively) to the binding energy. The overall computed free energy is similar to WT, in agreement with binding affinities (Table 2; Tables S2 and S3).

In BH3C, the staple replaces Gly13 and Gln17, the location of the staple forces it to point into the MCL-1 surface creating a steric clash and thus a strain on the backbone of the BH3C peptide which prevents the interaction with MCL-1, unlike with the other BH3 peptides.

In the wtBH3, Gln17 makes a strong hbond with Gly262 backbone, contributing ∼2 kcal/mol to the binding. This positions the staple (in place of Gly13-Gln17) into a potential clash between the peptide and MCL-1. This is alleviated by a conformational rearrangement such that Asp14 pulls away from Arg263, and forms a salt bridge with Arg18. The net result is increased strain in the peptide, helical conformation in the stapled region and poor helical content in the terminal regions (Figure S11D). The loss of key hbond networks result in decreased contributions from Arg10 (−3.6) and Asp14 (−0.1), when compared with BH3wt peptide (Figures 4C and 4D; Movies S3 and S4). The overall binding energy of BH3C peptide is reduced (∼16 kcal/mol) significantly compared with BH3wt (Table 2; Tables S2 and S3).

In BH3D, the staple bridging positions 17 and 21 results in a better overall helicity with retention of key interactions, with a part of the staple draped over MCL-1. In the wtBH3 sidechain of Gln17 makes a strong hbond with the Gly262 backbone; while sidechain of Glu21 interacts with the sidechain of Gln17. Replacing these residues with the staple derives additional hydrophobic contacts from neighboring residues Asn260, Trp261, Gly262, Phe318 and Phe319. The overall mobility of the peptide is significantly reduced in the C-terminal region (Figures 3A and B; Movies S5 and S6).

In the BH3D peptide, significant contributions were made by key hydrophobic residues i.e., Leu6, Leu9, Val12 and Val16 (−4.7, −4.8, −3.1 and −2.8 kcal/mol respectively), and also by polar residues i.e., Arg10 and Asp14 (−5.3 and −2.8 kcal/mol respectively) to the binding energy. The hydrocarbon staple contributes significantly (−4.3 kcal/mol), equivalent to that of Leu6 and Leu9. The contribution of the staple is highest among all the peptides. The contribution of Gln17 in BH3 wt is −1.7 kcal/mol, clearly suggesting that the staple contributes an extra ∼2.5 kcal/mol (Table 2; Tables S2 and S3).

The BH3E staple stabilizes the C-terminal region by reducing its mobility. However this staple is less packed against the surface of MCL-1 compared to the BH3D staple. The position of the BH3E enables Gln17 to make hbond interactions with the Gly262 backbone (Figures S14C and D). Significant contributions were made by key hydrophobic residues i.e., Leu6, Leu9, Val12 and Val16 (−5.3, −5.0, −3.0 and −3.2 kcal/mol respectively), and also by polar residues i.e., Arg10 and Asp14 (−4.5 and −3.2 kcal/mol respectively) to the binding energy (Table 2 and Tables S2 and S3). The hydrocarbon staple contributes an excess (−1.0 kcal/mol) over residues it replaces in BH3wt.

In conclusion, we find that amongst the peptides designed by Walensky et al. [24], the tightest binder BH3D retains the hbond networks that are characteristic of the interactions of the wild type (except for the hbond between the sidechain of Gln17 with the backbone of Gly262) and its staple packed against the MCL-l surface when compared with other staples reported.

### Designing New Peptides with Higher Affinity

#### Computational alanine scanning

Guided by the above findings, we next attempt to design peptides with higher affinities. We first use computational alanine scanning [59], on BH3wt whereby the orientation of the mutated sidechain is energetically optimized using SCWRL [69], [70], and the effects of this on the structure and interactions of MCL-BH3wt are computed. We also subject these calculations to 1 ns simulations each; however the simulations did not converge, so before investing in longer simulations, we decided to use the results arising from energy minimizations. L6A, L9A and V16A mutations were found to have reduced affinity for MCL-1 by 3-5 kcal/mol (Table S4), reflecting the importance of the larger sidechains Val and Leu in the hydrophobic interactions with the surface. In addition, R10A and D14A also were associated with loss in binding affinity because of the loss in their hbond networks. This motivated us to vary the staple points across the other residues as their changes do not seem to perturb the affinity.

#### New staple positions and mutations

We noticed that in all the simulations, Arg18 interacts with both Glu21 and Thr22 transiently. So we reasoned that mutation of Thr22 to Asp may enhance the stability by forming a salt bridge between Arg18 and Asp22. This peptide, called BH3F (Figures S14E and F) did not yield any improvements because the charged residues only interacted transiently and preferred to remain solvated (Table 2; Tables S2 and S3); some reduction in mobility at the C-terminus was evident.

We modified BH3B (the staple is across positions 7 and 11) and added an extra staple that linked the Arg18 and Thr22 positions at the C-terminus; double stapling has been used successfully in the context of longer peptides [71]. In this peptide, called BH3G, both staples remain solvent exposed, and did not interact significantly with the MCL surface. However, the staples stabilize the helicity when compared with the wt type MCL-1. The hbond clusters between the peptide and the protein were well maintained (Figures S14G and H). In the BH3G peptide, significant contributions were made to the overall binding energy of −26.4 kcal/mol by hydrophobic residues i.e., Leu6, Leu9, Val12 and Val16 (−5.3, −4.8, −3.2 and −2.7 (kcal/mol) respectively) and also by polar residues i.e., Arg10 and Asp14 (−5.1 and −3.2 (kcal/mol) respectively) to the binding energy. In addition, the N-terminal staple, being closer to the surface of MCL-1, contributed −3.1 kcal/mol whereas the C-terminal staple contribution was negligible (−0.4 kcal/mol).

In contrast to the observation that the key hydrophobic residues Leu6, Leu9 and Val16 present in the BH3 peptides are embedded into the hydrophobic pocket on the surface of MCL-1, Val12 is partially exposed. Val12 is surrounded by His224, Phe228, Met231 and Phe270 which is a hydrophobic patch and hence offers an opportunity for exploitation by the introduction of a staple in the vicinity of these residues. To explore this, we took BH3D and added a second staple that linked Thr8 with Val12 (called BH3H). The binding affinity of this peptide improved by ∼7 kcal/mol compared to BH3D and mostly arose from the improved packing of this stapled region against His224, Phe228, Met231 and Phe270. Our simulations suggest that this region offers a well defined hydrophobic patch (Figures 4E and 4F; Movies S7 and S8). The overall binding energy (−33.4 kcal/mol) comprises contributions from hydrophobic residues i.e., Leu6, Leu9 and Val16 (−3.8, −4.5 and −3 (kcal/mol) respectively) and also by polar residues i.e., Arg10 and Asp14 (−5.9 and −3 (kcal/mol) respectively). The N-terminal staple that was introduced, contributed −7.3 kcal/mol (the highest so far amongst the staples) while the C-terminal staple (BH3D staple) contributed −4.5 kcal/mol. Together these two staples contribute ∼33% of the overall binding energy (Table 2; Tables S2 and S3).

To further optimize BH3H, we noticed that Ala5 lies in the vicinity of Lys234 (Figures 4E and F) and so we mutated it to Asp5 (called BH3I) to introduce a potential salt bridge. We further mutated Arg18 to Asp (called BH3J) in order to reduce the mobility at the C-terminus. However, the Asp5-Lys234 interaction was only transient in both BH3I and BH3J, but the mobility of BH3J was reduced. The associated binding energies were −33.5 and −31.7 kcal/mol respectively (Table 2; Tables S2 and S3). Clearly these changes did not result in any significant differences in the affinity compared to BH3H (Figures S14 I, J, K and L).

We finally took BH3H and removed the C-terminal staple (the one introduced by Walensky in BH3D) to examine the interactions of the peptide with only an N-terminal staple. We find that this did not disturb the hbond cluster between the protein and the peptides (Figures 4G and 4H; Movies S9 and S10). Indeed, the removal of the BH3D staple brings back Gln17 which stabilizes the system by hbonding to the backbone of Gly262, as in the wild type system. The overall binding energy surprisingly remains −33.7 kcal/mol and contributions from hydrophobic residues i.e., Leu6, Leu9 and Val16 are −5.5, −4.5 and −2.9 kcal/mol respectively while those from polar residues i.e., Arg10 and Asp14 are −5.8 and −3.2 kcal/mol respectively. The staple contributed −7.4 kcal/mol, as in BH3H. This clearly shows that the effects of the staples at the two termini are decoupled from each other.

The staple connecting Gln17-Glu21 in BH3D contributes −4.3 kcal/mol to the binding, while in wild type, the contribution of Gln17 is −1.7 kcal/mol. Thus the net contribution of this staple is ∼2.5 kcal/mol. However, in BH3K (or indeed in BH3H), the staple connecting Thr8-Val12, contributes ∼7.3 kcal/mol which is much higher (the contributions of Thr8 and Val12 are −0.4 and −3.2 kcal/mol, totaling ∼3 kcal/mol less than the staple that replaces them). Moreover this peptide also has the Gln17 sidechain making hbond with Gly262, and contributes ∼2 Kcal/mol to binding energy (Table 2; Tables S2 and S3).

### Conclusions

Inhibition of protein-protein interactions and modulation of associated signaling is slowly gaining popularity as progress is made in areas of fragment based drug discovery [72], [73], peptidomimetics [74] etc. The latest addition to this collection, that is demonstrating great promise, are stapled peptides [24], [28], [32]. So far, these have been most effective in targeting proteins where the target site requires a helical motif in its binding partner. Stapled peptides appear to be excellent at this since they already are preorganzied into a helical fold, thus reducing the entropic costs of localization [24], [28], [32], [33].

Walensky et al. [24], have optimized the BH3 helix through stapling as a potent MCL-1 inhibitor. They demonstrated through structural studies that the staple derives additional binding energy by interacting with a hydrophobic patch on the MCL-1 surface. MD simulations of these peptides show that (a) the location of the staple is a key determinant of good from bad binder. (b) the good binders derive binding affinity from interactions between the hydrophobic staple and hydrophobic patches on the MCL-1 surface; indeed the contribution to the binding energies due to these interactions can be as large as that contributed by the buried residues (c) there are peptides that bind with higher affinity but the staples appear to point out into solvent (BH3A/BH3B); these staples seem to “push” the peptide into the binding site, yielding tighter interactions of the buried residues (Table S3) and are similar to observations made elsewhere [75]; indeed, stapled peptides against the HIV capsid protein have also been shown by NMR to have the staples pointing into solvent [36]; the observation that BH3C is most helical in solution and yet the worst binder appears paradoxical at first glance and yet upon scrutiny reminds and educates us that the interaction between protein and peptide is modulated by very dynamic surfaces [41], [76] and that perhaps creation of too tight a helix in the peptide in solution will hinder an efficient capture and binding of the peptide by the target protein surface. Clearly more detailed studies on diverse systems will appear in the near future and give us glimpses into structure-activity relationships between the amino acid compositions of peptides, the optimal locations of staples, the ability to enter cells unaided and the mechanisms of these exciting molecules which hold promise as a new class of reagents for interrogating biology and as therapeutics. For now, guided by the findings of Walensky and his group, and the insights offered by the MD simulations, we have carried out mutagenesis to design peptides that computationally demonstrate higher affinities for MCL-1 and are currently being tested in the laboratories of collaborators.

## Supporting Information

Figure S1 **BH3A bound to MCL-1 (shown in grey).** (A) R10 sidechain makes hbonds with the H252 backbone and the S255 sidechain, while the D14 sidechain makes hbonds with the sidechains of N260 and R263 (shown in cartoon), (B) The hydrophobic residues are deeply buried inside MCL-1 (shown in surface).
(PDF)

Figure S2 **Root mean squared deviation for the MCL-1 in their bound form with the BH3 peptides.**
(PDF)

Figure S3 **Root mean squared deviation for the BH3 peptides in their bound form with MCL-1.**
(PDF)

Figure S4 **Root mean squared deviation for the BH3 peptides in solution.**
(PDF)

Figure S5 **Radius of gyration for the MCL-1 in complex with BH3 peptides.**
(PDF)

Figure S6 **Radius of gyration for the BH3 peptides in complexes.**
(PDF)

Figure S7 **Radius of gyration for the BH3 peptides in solution.**
(PDF)

Figure S8 **Root mean squared fluctuations for the BH3 peptides in solution.**
(PDF)

Figure S9 **Temporal evolution of the secondary structure profiles of BH3 peptides over 20 ns in solution (A) BH3wt; (B) BH3A; (C) BH3B; (D) BH3C; (E) BH3D; (F) BH3E; (G) BH3F; (H) BH3G; (I) BH3H; (J) BH3I; (K) BH3J and (L) BH3K.** It is clear that the wild type peptide assumes a largely helical conformation in solution, especially in the Leu6–Gln17 region.
(PDF)

Figure S10 **Solution structures of unbound the BH3 peptides.** (A) Helicity is observed in the regions of Leu6–Glu21 in BH3wt. (B) BH3C peptide is the most helical among all the unbound peptides analyzed, and interestingly is also the only inactive peptide and (C) Helicity in the BH3D peptide extends from Leu6–Gln17 (crystallographically observed) to Leu6–Glu21.
(PDF)

Figure S11 **Temporal evolution of the secondary structure profiles of the BH3 peptides when bound with MCL-1 over 20 ns in solution.** (A) BH3wt; (B) BH3A; (C) BH3B; (D) BH3C; (E) BH3D; (F) BH3E; (G) BH3F; (H) BH3G; (I) BH3H; (J) BH3I; (K) BH3J and (L) BH3K. When complexed to MCL-1, all peptides except BH3C are helical, especially in the Glu7–Thr22 region.
(PDF)

Figure S12 **Root mean squared fluctuations for MCL-1 in complex with the BH3 peptides.**
(PDF)

Figure S13 **Root mean squared fluctuations for the BH3 peptides in complexes.** BH3C peptide show higher fluctuations when compared with all other peptides.
(PDF)

Figure S14 **BH3B bound to MCL-1 (shown in grey).** (A) Asp14 maintains the hbond network with Arg263, whilst Gln17 makes an hbond interaction with Gly262. (shown in cartoon), (B) The hydrophobic groups Leu6, Leu9, and Val16 are buried in the hydrophobic binding groove on the surface of MCL-1 (shown in surface); BH3E bound to MCL-1 (shown in grey) (C) BH3E staple is less packed against the surface of MCL-1 compared to the staple in the best binder BH3D, but the positioning of the staple enables Gln17 to make hbond interactions with the Gly262 backbone (shown in cartoon), (D) The hydrophobic groups Leu6, Leu9, and Val16 are buried in the hydrophobic binding groove on the surface of MCL-1 (shown in surface); BH3F bound to MCL-1 (shown in grey) (E) Interactions similar to those made by BH3D are also observed in the BH3F peptide bound to MCL-1, with the staple enabling Gln17 to make hbond interactions with the Gly262 backbone (shown in cartoon), (F) The hydrophobic groups Leu6, Leu9, and Val16 are buried in the hydrophobic binding groove on the surface of MCL-1 (shown in surface); BH3G bound to MCL-1 (shown in grey) (G) Double stapling improves the packing of the stapled regions and also maintains the helical content (shown in cartoon), (H) The hydrophobic groups Leu6, Leu9, and Val16 are buried in the hydrophobic binding groove on the surface of MCL-1 (shown in surface); BH3I bound to MCL-1 (shown in grey) (I) Double stapling improves the packing of those stapled regions and also maintains the helical content (shown in cartoon), (J) The hydrophobic groups Leu6, Leu9, and Val16 are buried in the hydrophobic binding groove on the surface of MCL-1 (shown in surface); BHJ bound to MCL-1 (shown in grey) (K) Double stapling improves the packing of the stapled regions and also maintains the helical content (shown in cartoon), (L) The hydrophobic groups Leu6, Leu9, and Val16 are buried in the hydrophobic binding groove on the surface of MCL-1 (shown in surface).
(PDF)

Table S1 **Binding enthalpies (kcal/mol) of BH3A stapled peptide against MCL-1 using single point computational alanine scanning.**
(PDF)

Table S2 **Components of binding free energy (in kcal/mol) of MCL-1 with BH3 peptides.**
(PDF)

Table S3 **Residuewise energy contributions (in kcal/mol) of BH3 peptides for its interactions with MCL-1.**
(PDF)

Table S4 **Binding enthalpies (kcal/mol) of BH3-wt peptide against MCL-1 using single point computational alanine scanning.**
(PDF)

Movie S1 **Movie of MD simulation trajectory of BH3wt bound to MCL-1 (MCL-1 is shown in cartoon).**
(WMV)

Movie S2 **Movie of MD simulation trajectory of BH3wt bound to MCL-1(MCL-1 is shown in surface).**
(WMV)

Movie S3 **Movie of MD simulation trajectory of BH3C bound to MCL-1 (MCL-1 is shown in cartoon).**
(WMV)

Movie S4 **Movie of MD simulation trajectory of BH3C bound to MCL-1 (MCL-1 is shown in surface).**
(WMV)

Movie S5 **Movie of MD simulation trajectory of BH3D bound to MCL-1 (MCL-1 is shown in cartoon).**
(WMV)

Movie S6 **Movie of MD simulation trajectory of BH3D bound to MCL-1 (MCL-1 is shown in surface).**
(WMV)

Movie S7 **Movie of MD simulation trajectory of BH3H bound to MCL-1 (MCL-1 is shown in cartoon).**
(WMV)

Movie S8 **Movie of MD simulation trajectory of BH3H bound to MCL-1 (MCL-1 is shown in surface).**
(WMV)

Movie S9 **Movie of MD simulation trajectory of BH3K bound to MCL-1 (MCL-1 is shown in cartoon).**
(WMV)

Movie S10 **Movie of MD simulation trajectory of BH3K bound to MCL-1 (MCL-1 is shown in surface).**
(WMV)
