# Supplementary figures and images for: Stapled BH3 Peptides against MCL-1: Mechanism and Design Using Atomistic Simulations
Source: PLoS One. 2012 Aug 31;7(8):e43985. doi: 10.1371/journal.pone.0043985 (PMC3432064; doi:10.1371/journal.pone.0043985)

**Figure S1**

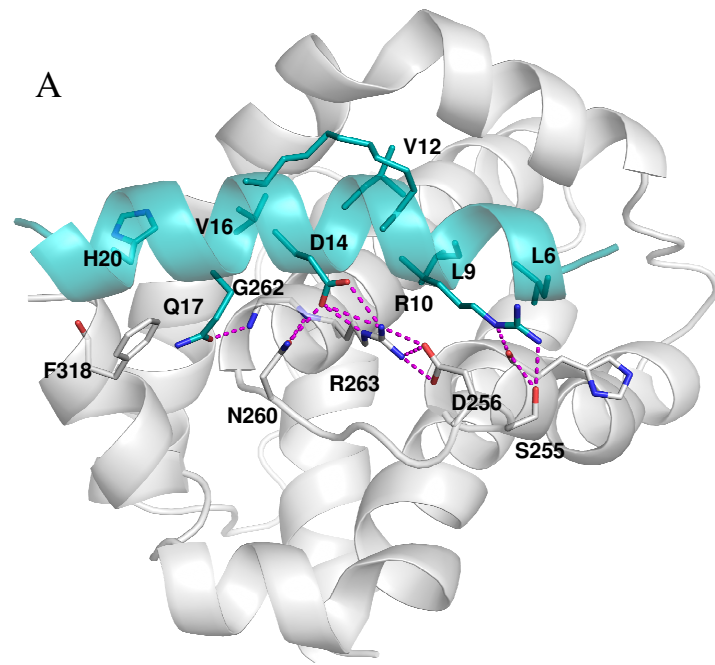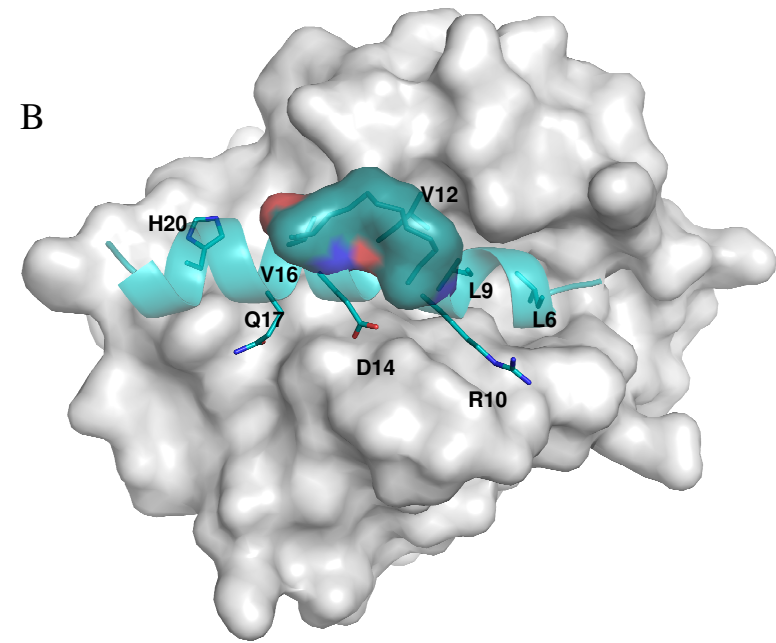

Supplement: Figure S1 — BH3A bound to MCL-1 (shown in grey). (A) R10 sidechain makes hbonds with the H252 backbone and the S255 sidechain, while the D14 sidechain makes hbonds with the sidechains of N260 and R263 (shown in cartoon), (B) The hydrophobic residues are deeply buried inside MCL-1 (shown in surface). (PDF) [file pone.0043985.s001.pdf]

**Figure S2**

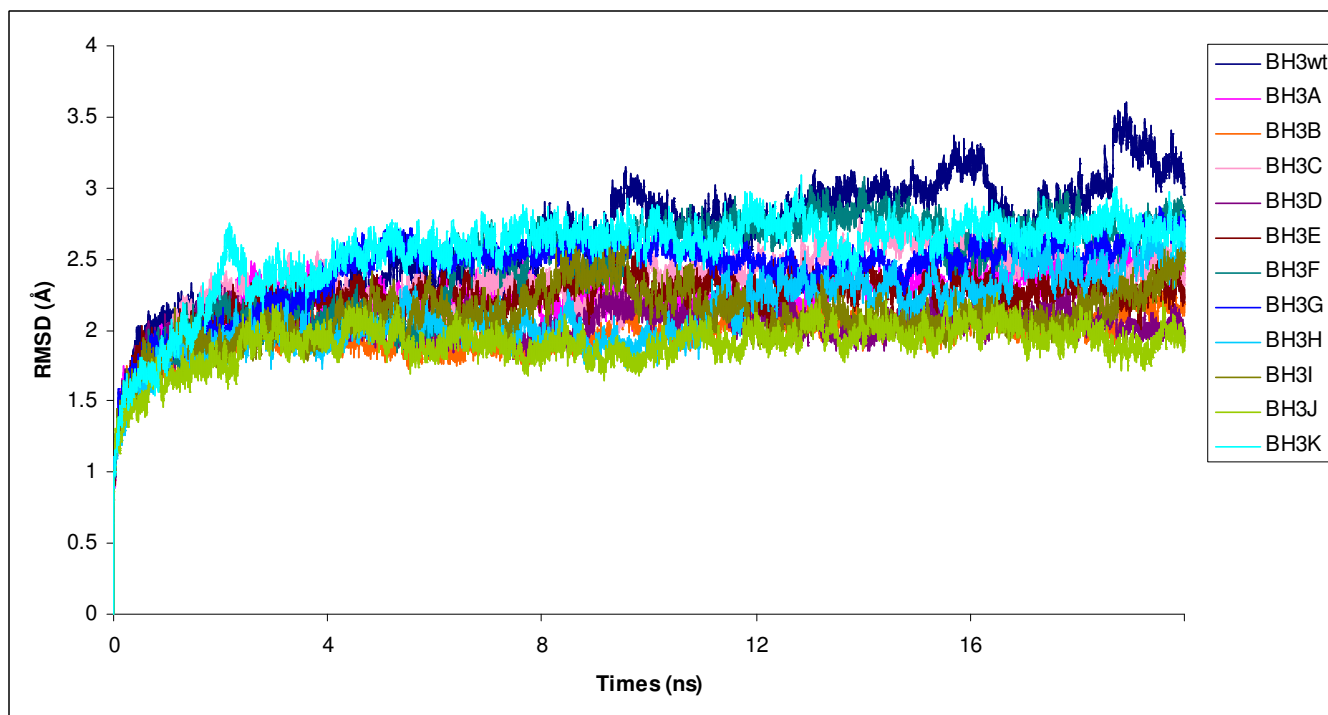

Supplement: Figure S2 — Root mean squared deviation for the MCL-1 in their bound form with the BH3 peptides. (PDF) [file pone.0043985.s002.pdf]

**Figure S3**

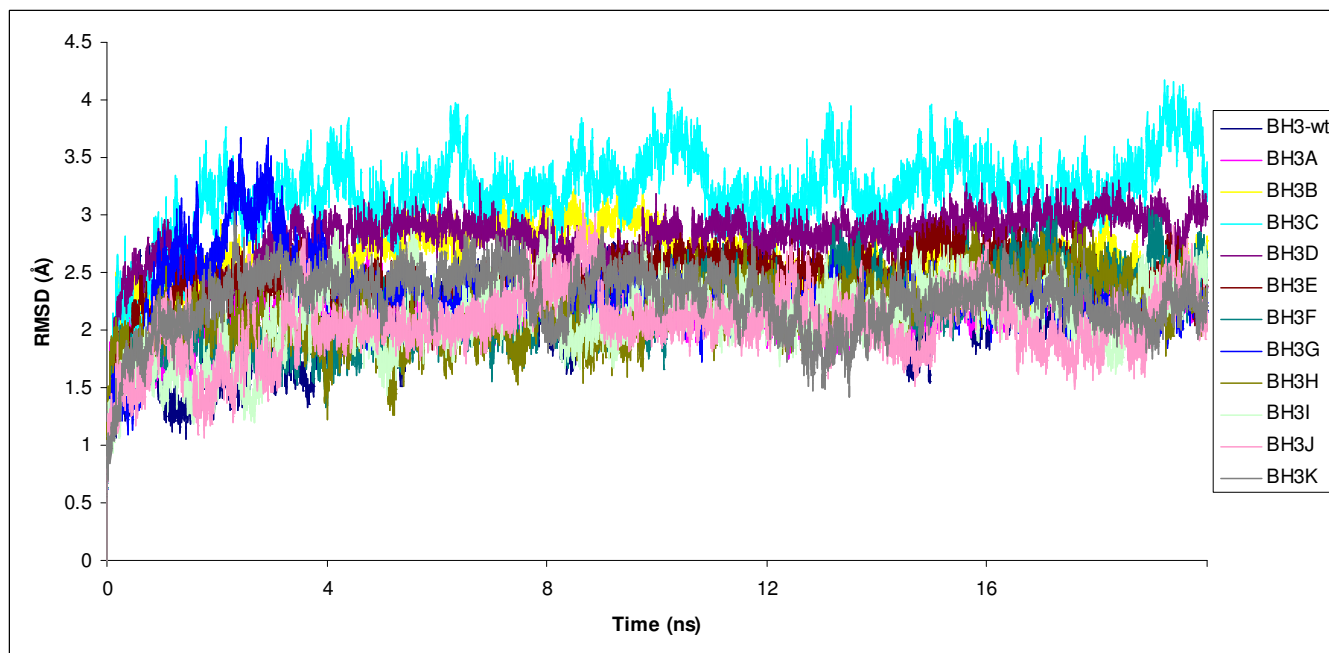

Supplement: Figure S3 — Root mean squared deviation for the BH3 peptides in their bound form with MCL-1. (PDF) [file pone.0043985.s003.pdf]

**Figure S4**

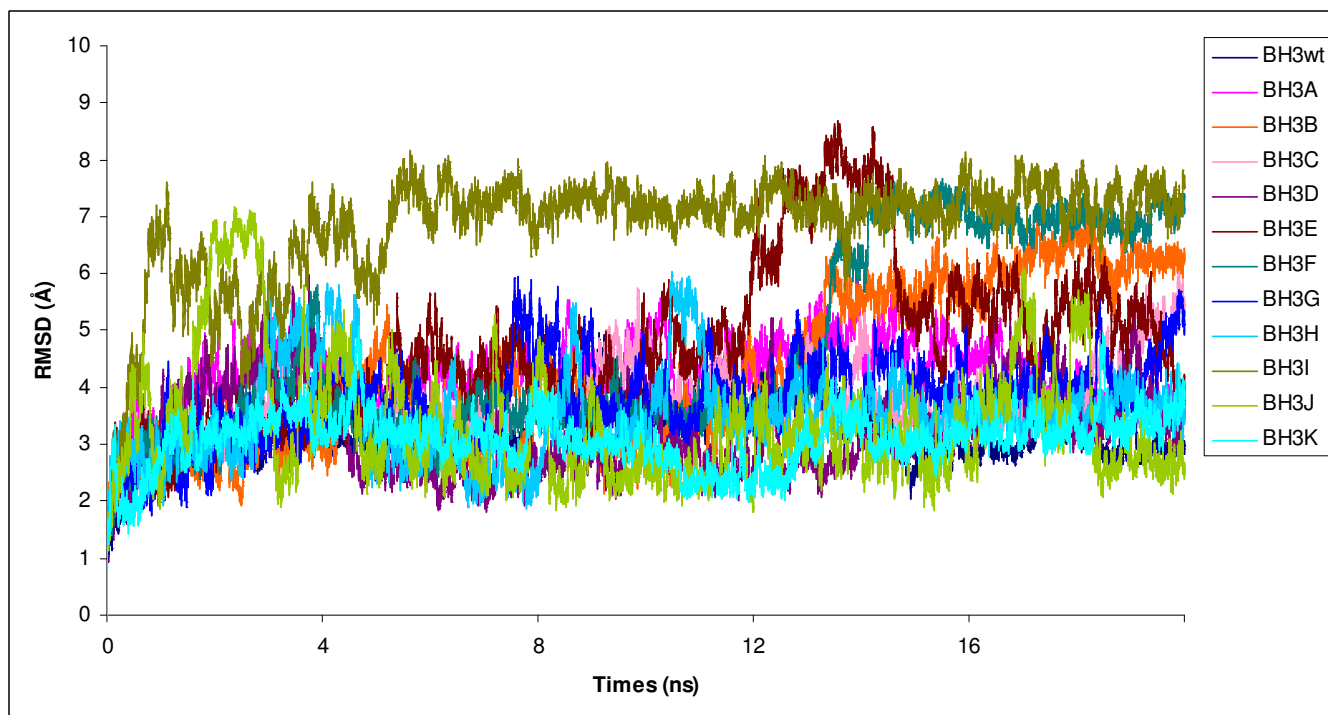

Supplement: Figure S4 — Root mean squared deviation for the BH3 peptides in solution. (PDF) [file pone.0043985.s004.pdf]

**Figure S5**

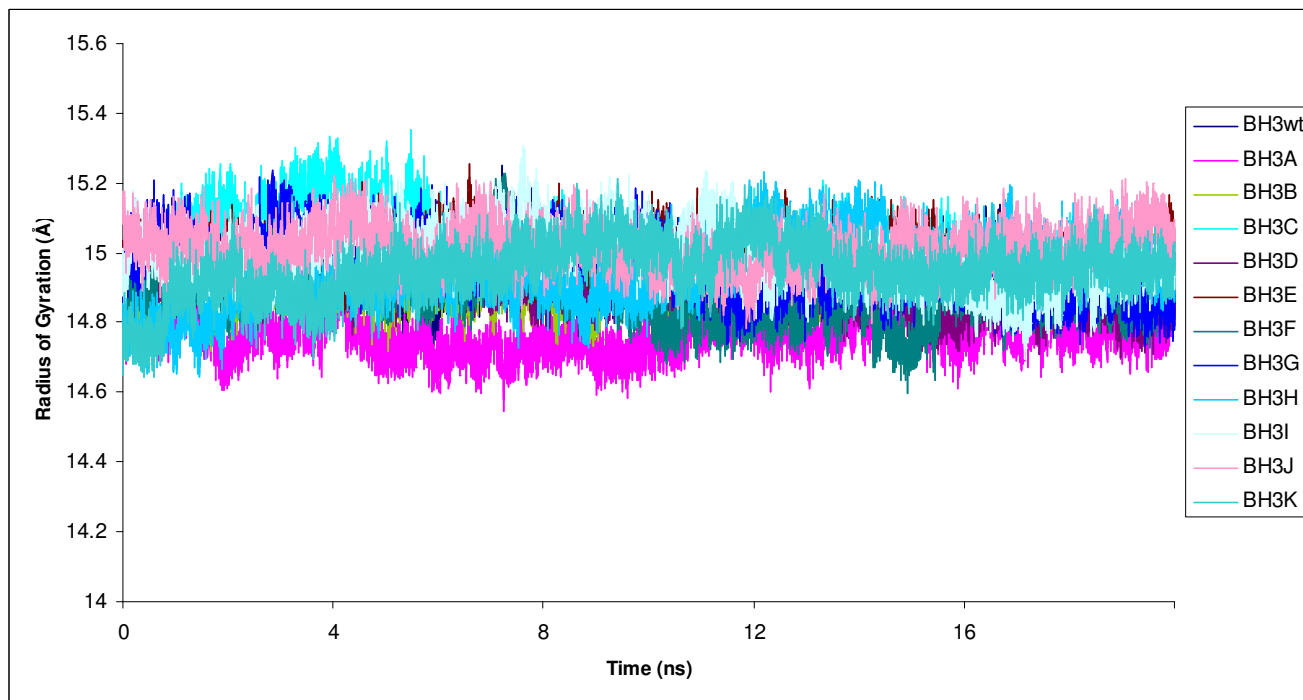

Supplement: Figure S5 — Radius of gyration for the MCL-1 in complex with BH3 peptides. (PDF) [file pone.0043985.s005.pdf]

**Figure S6**

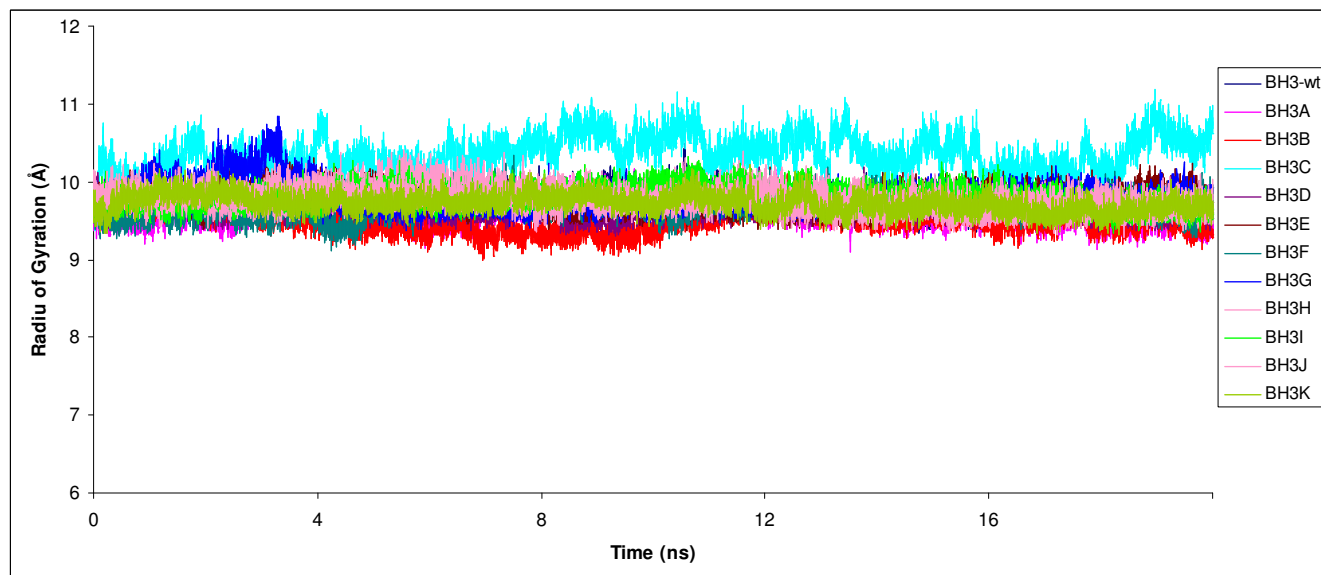

Supplement: Figure S6 — Radius of gyration for the BH3 peptides in complexes. (PDF) [file pone.0043985.s006.pdf]

**Figure S7**

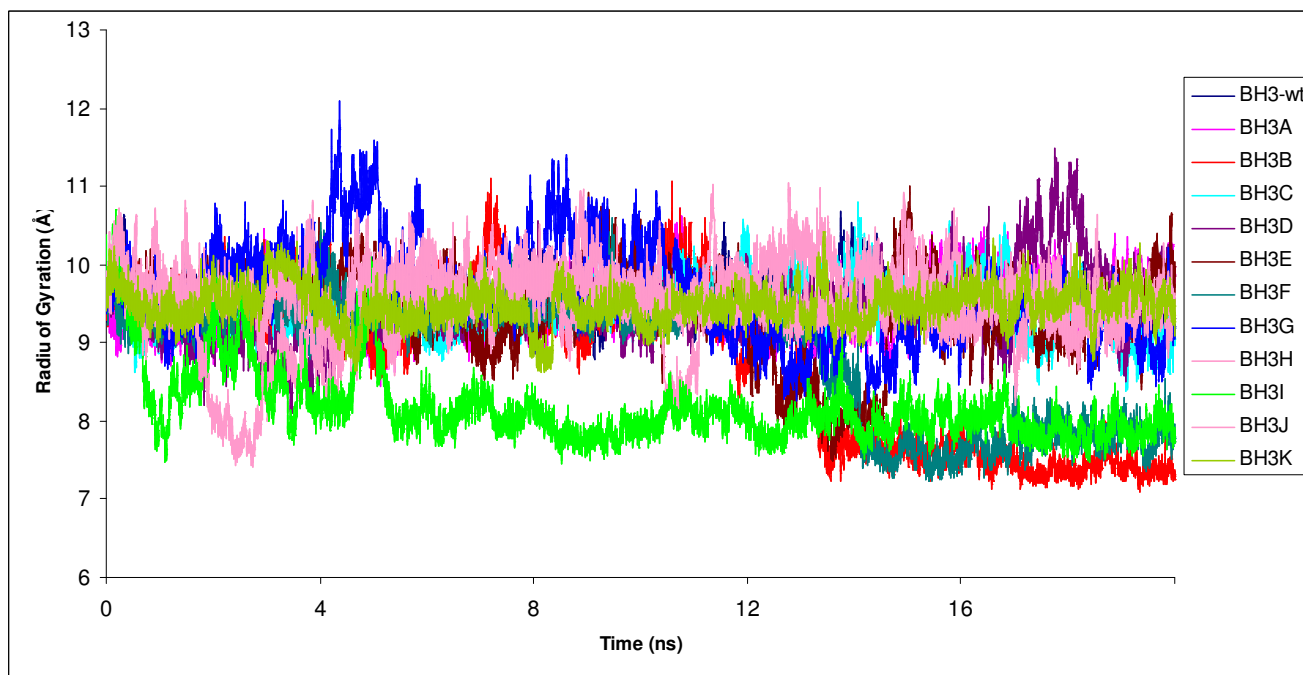

Supplement: Figure S7 — Radius of gyration for the BH3 peptides in solution. (PDF) [file pone.0043985.s007.pdf]

**Figure S8**

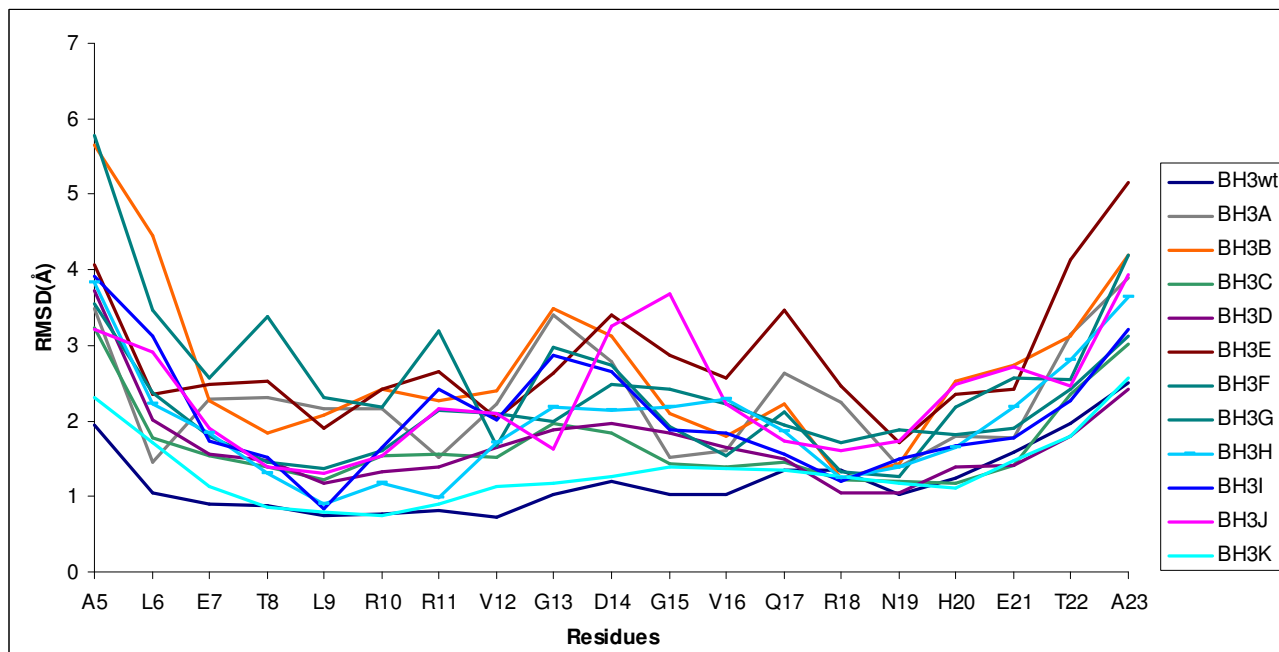

Supplement: Figure S8 — Root mean squared fluctuations for the BH3 peptides in solution. (PDF) [file pone.0043985.s008.pdf]

Figure S9 A

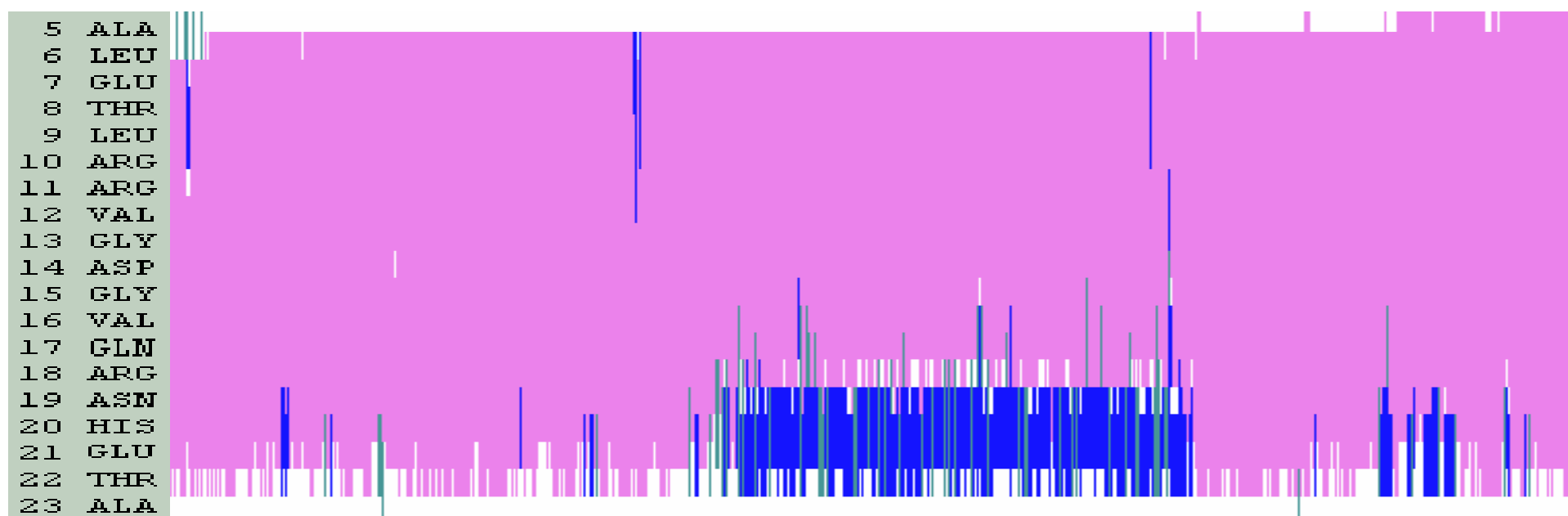

B

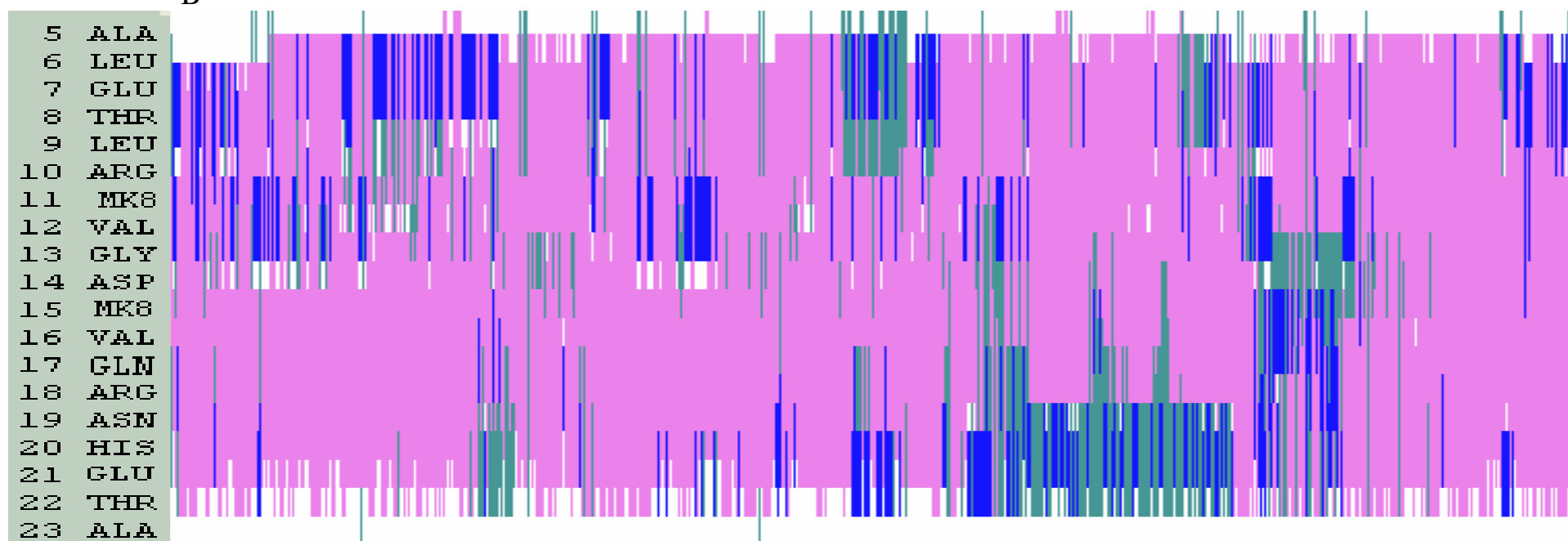

C

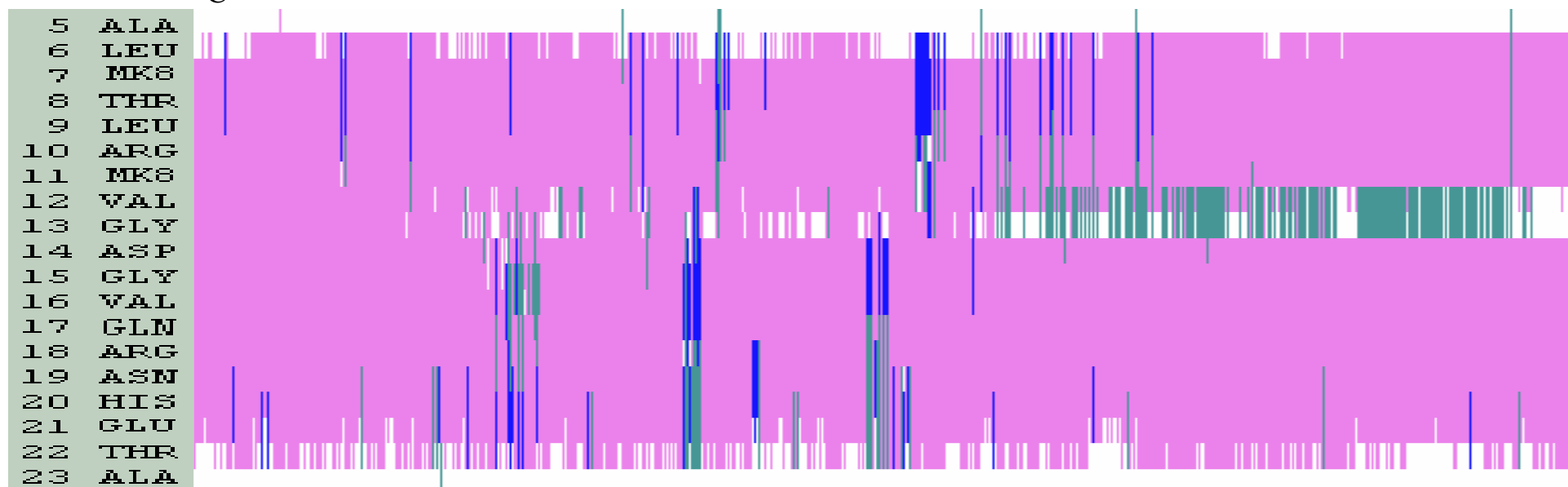

D

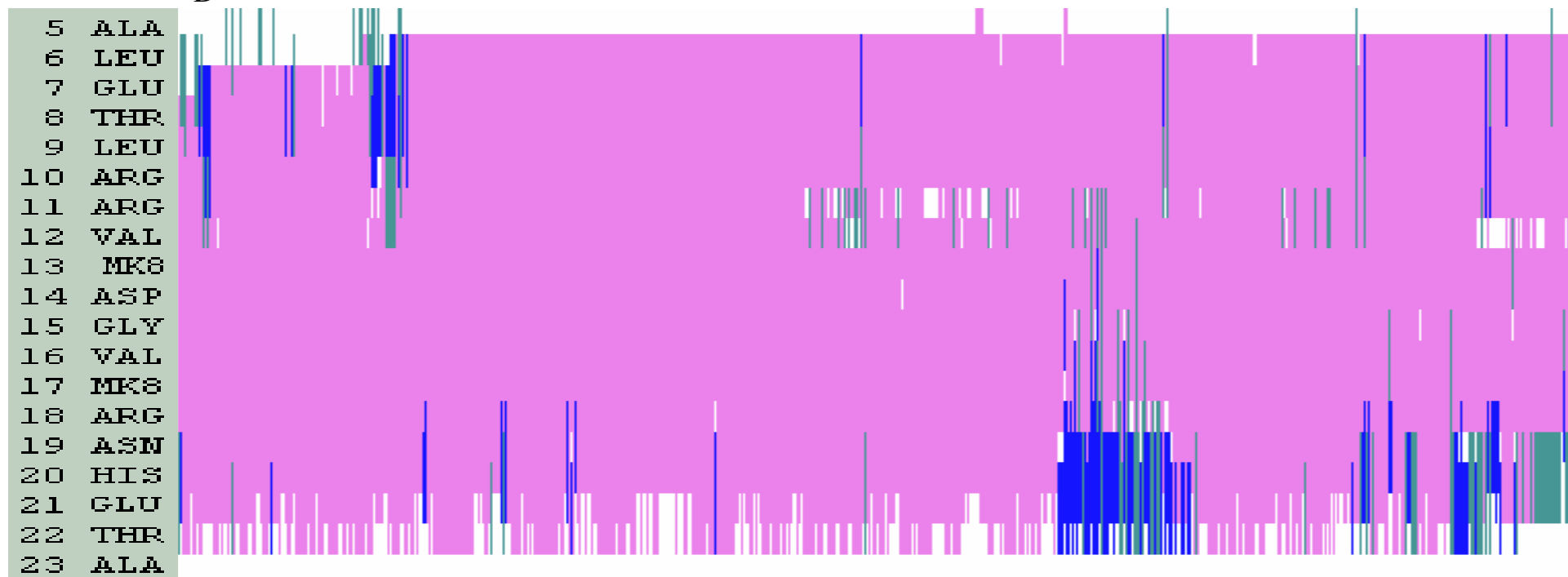

E

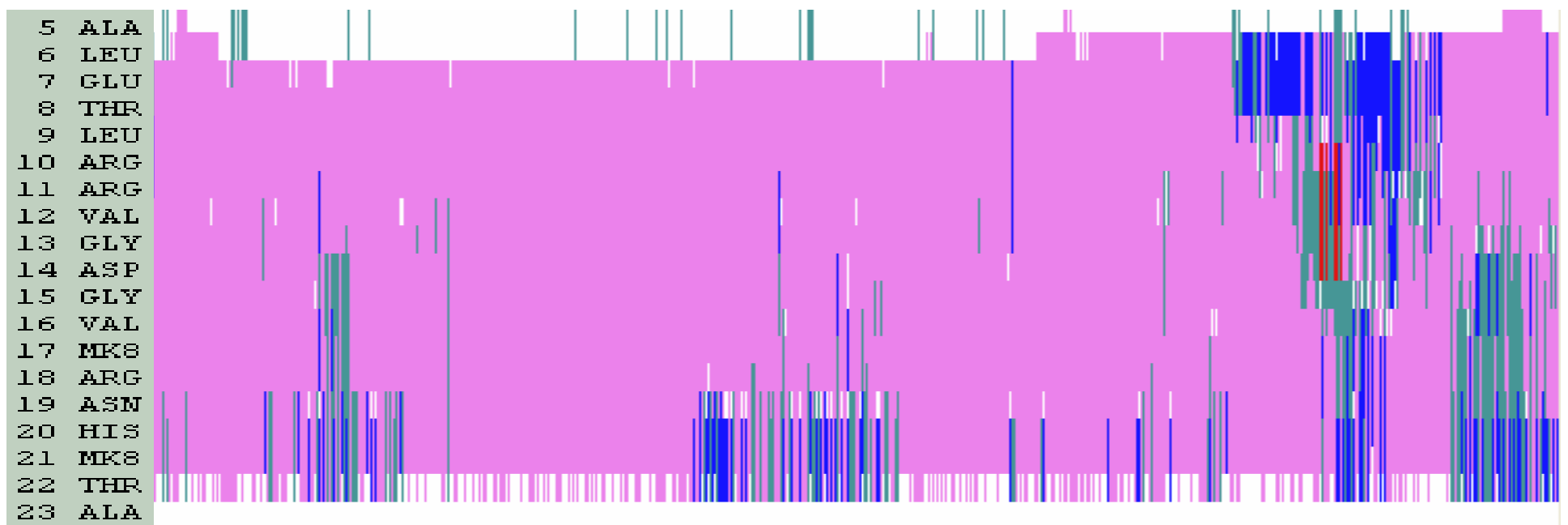

F

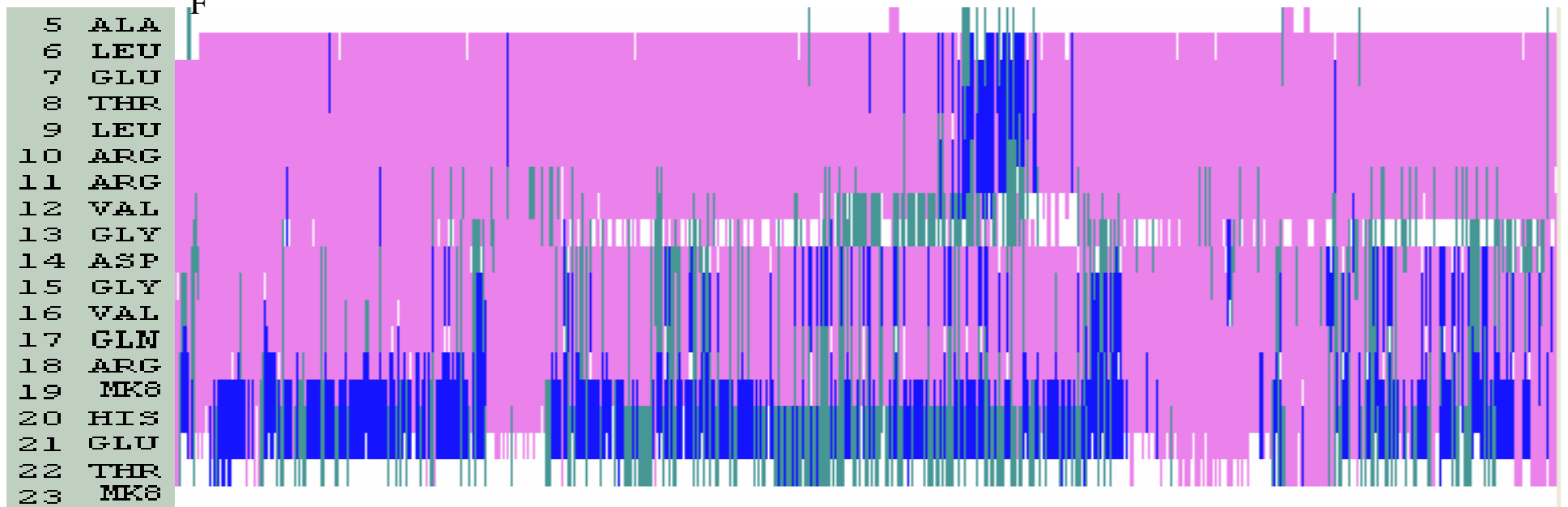

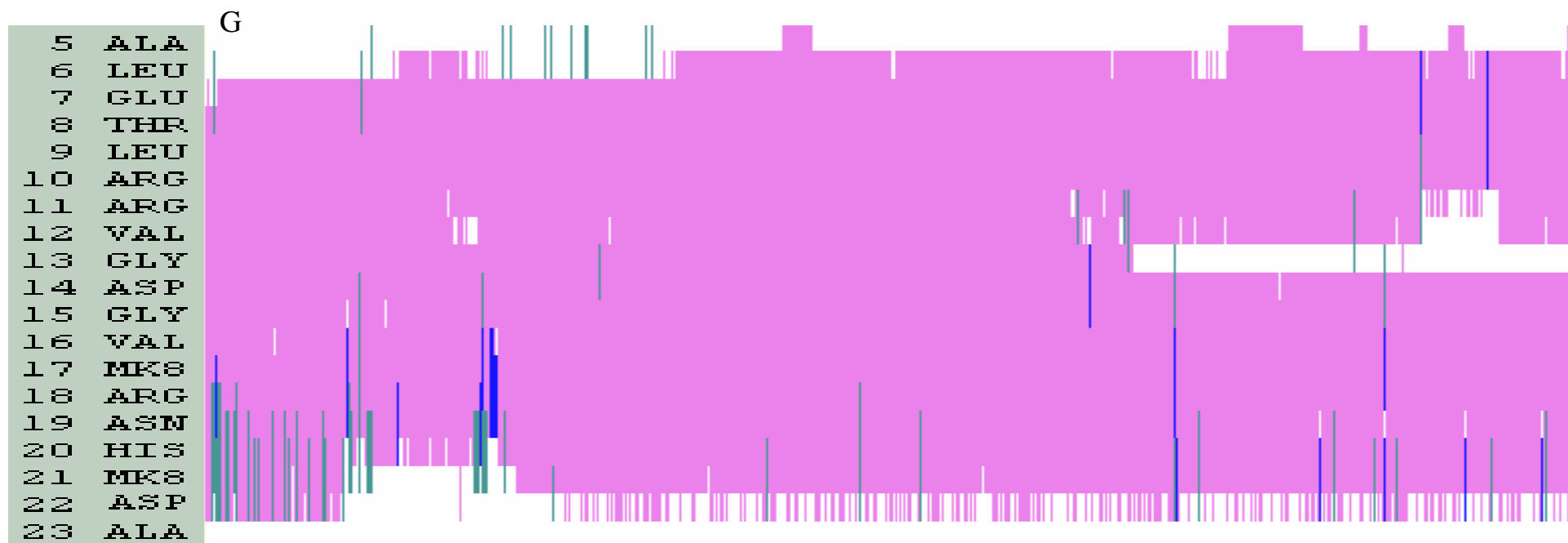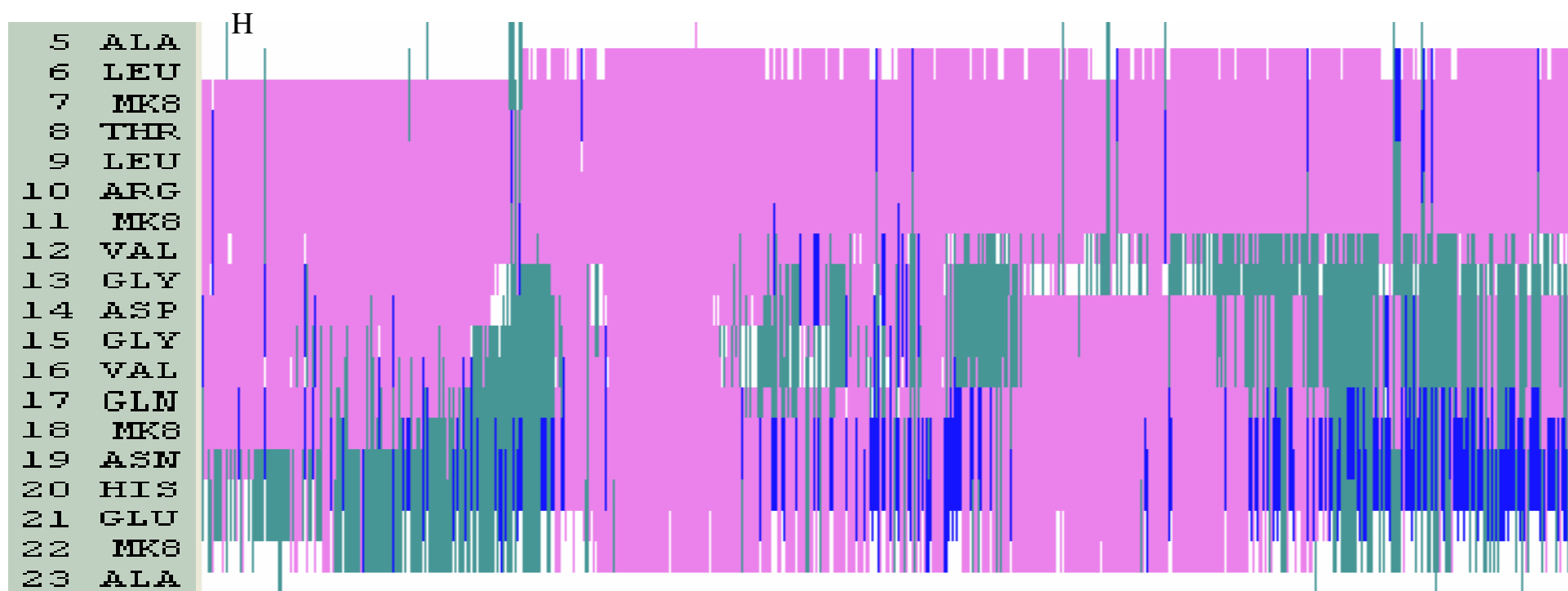

# I

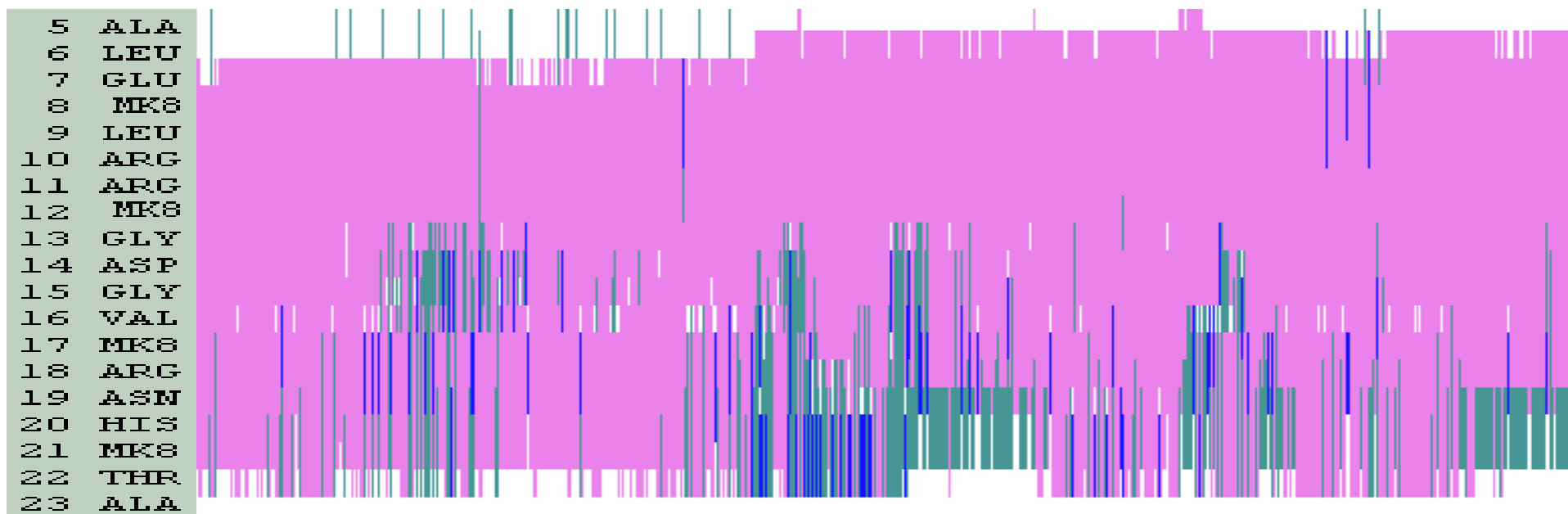

# J

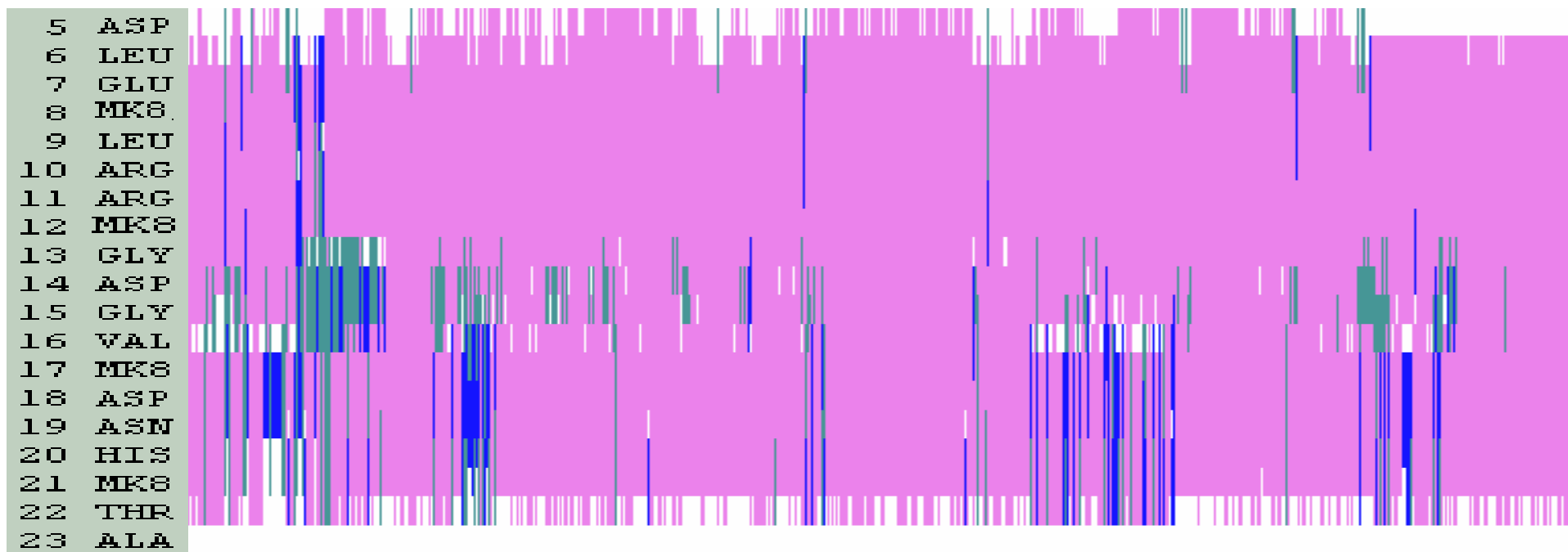

K

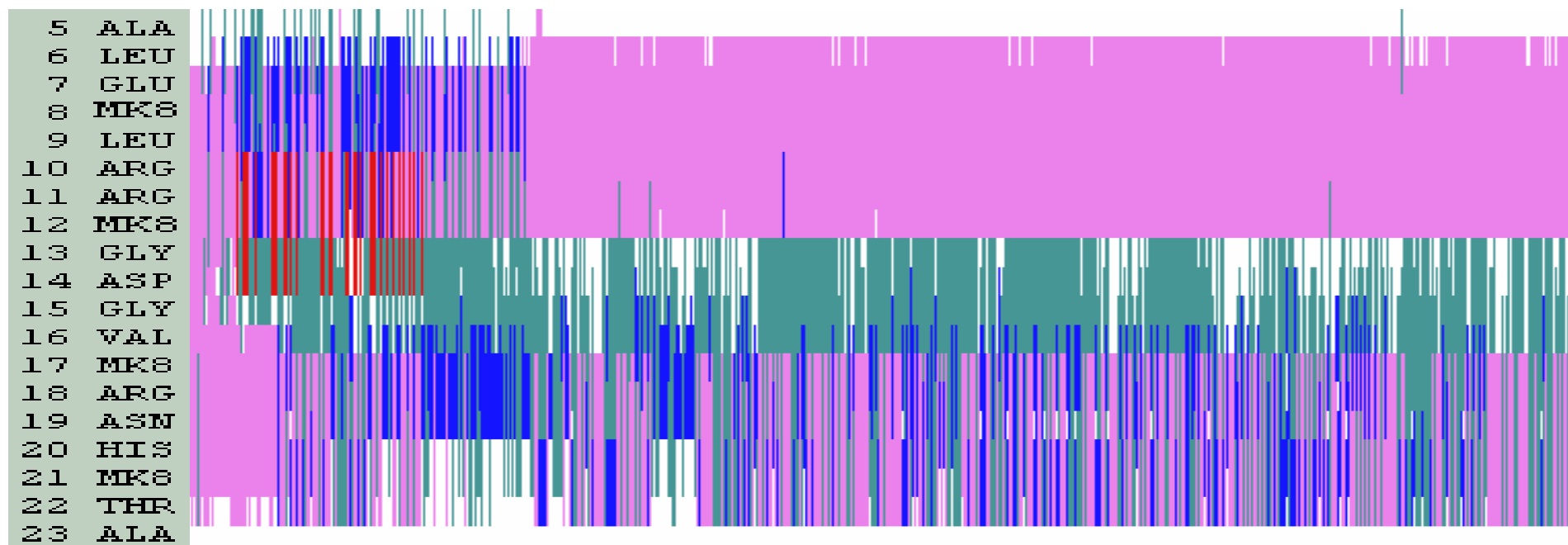

L

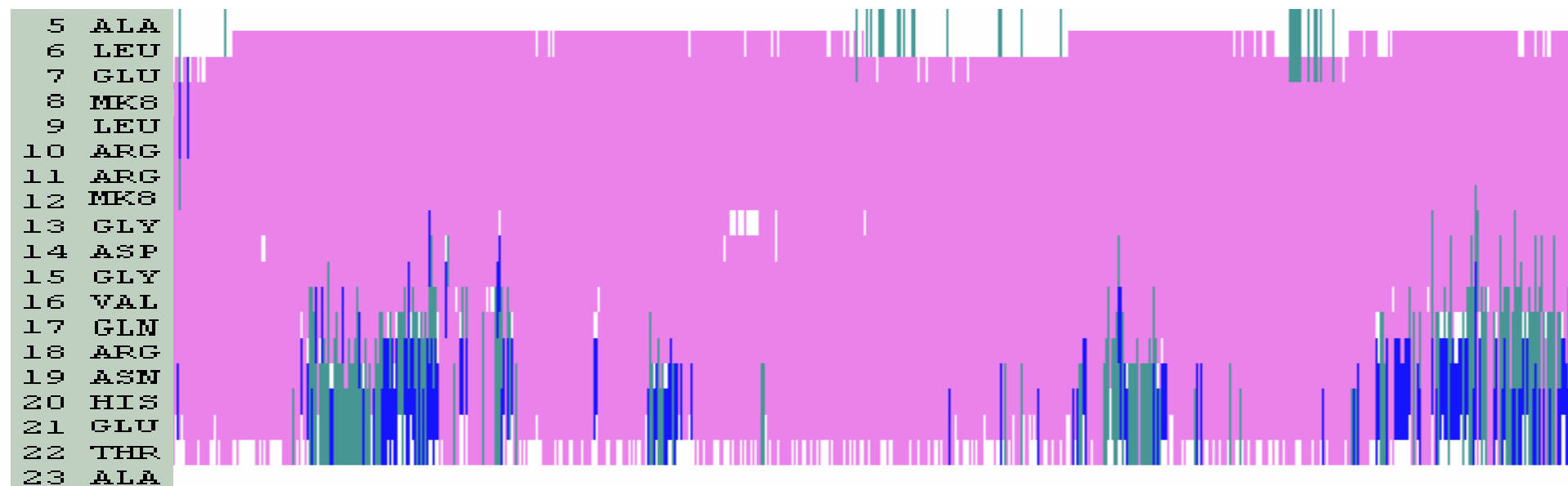

Supplement: Figure S9 — Temporal evolution of the secondary structure profiles of BH3 peptides over 20 ns in solution (A) BH3wt; (B) BH3A; (C) BH3B; (D) BH3C; (E) BH3D; (F) BH3E; (G) BH3F; (H) BH3G; (I) BH3H; (J) BH3I; (K) BH3J and (L) BH3K. It is clear that the wild type peptide assumes a largely helical conformation in solution, especially in the Leu6–Gln17 region. (PDF) [file pone.0043985.s009.pdf]

**Figure S10**

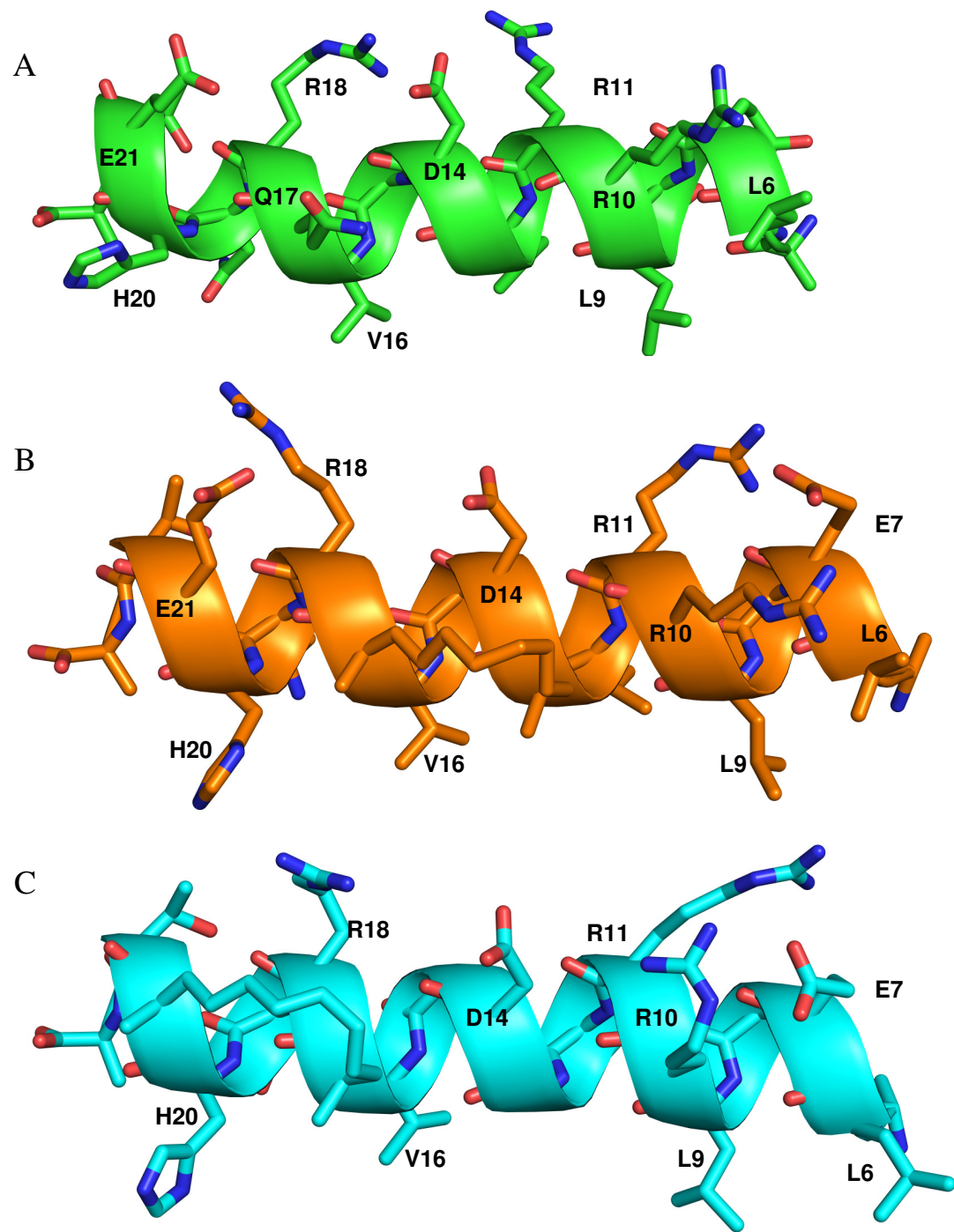

Supplement: Figure S10 — Solution structures of unbound the BH3 peptides. (A) Helicity is observed in the regions of Leu6–Glu21 in BH3wt. (B) BH3C peptide is the most helical among all the unbound peptides analyzed, and interestingly is also the only inactive peptide and (C) Helicity in the BH3D peptide extends from Leu6–Gln17 (crystallographically observed) to Leu6–Glu21. (PDF) [file pone.0043985.s010.pdf]

Figure S11 A

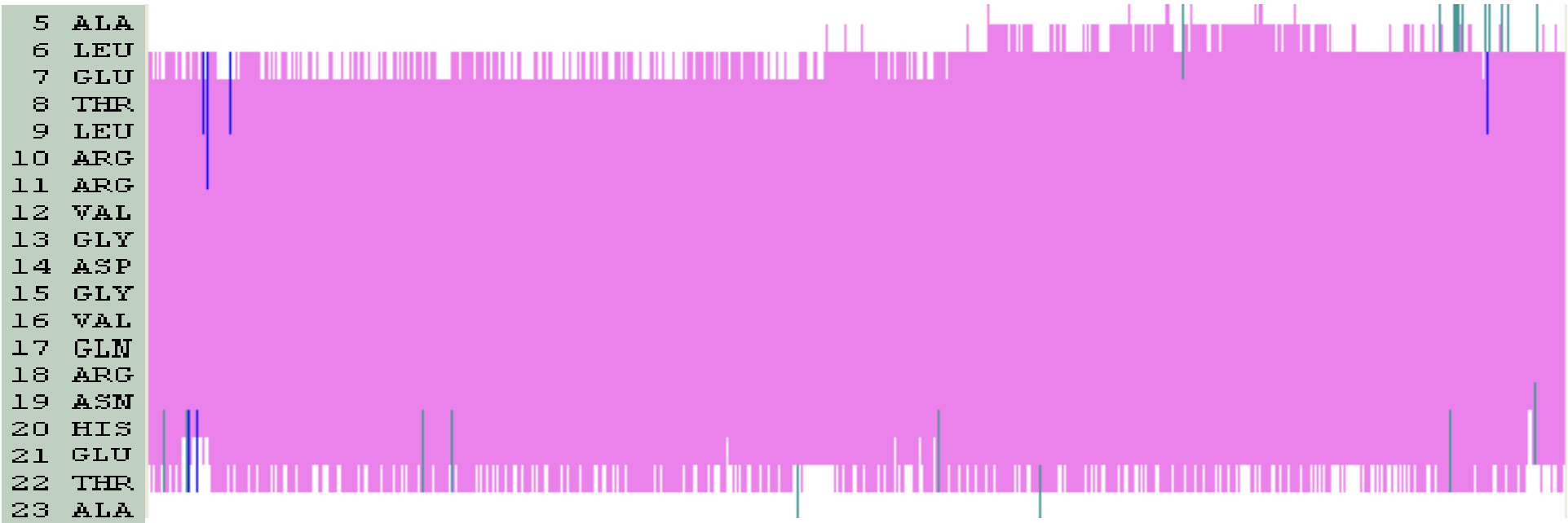

B

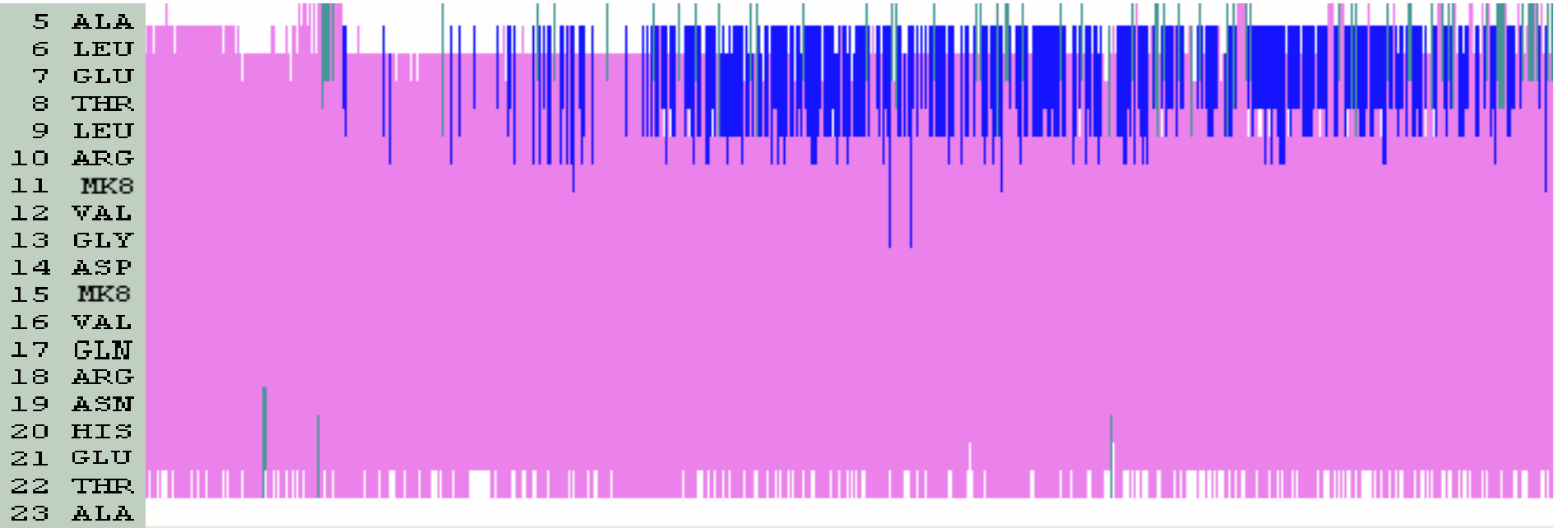

C

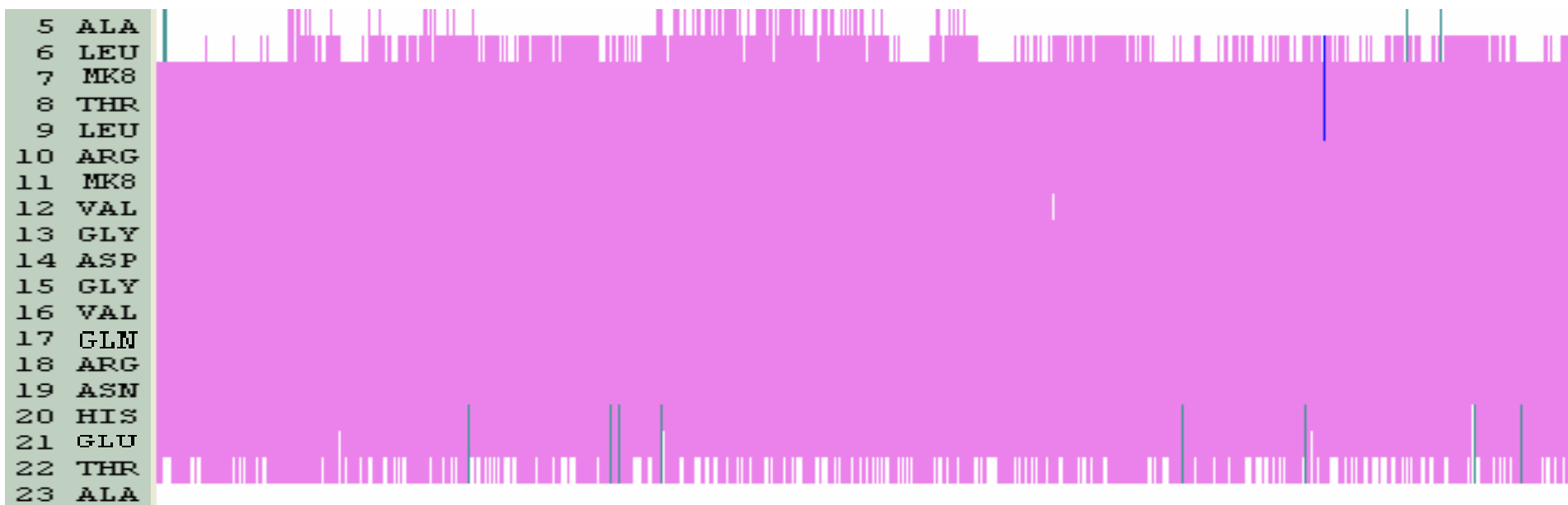

D

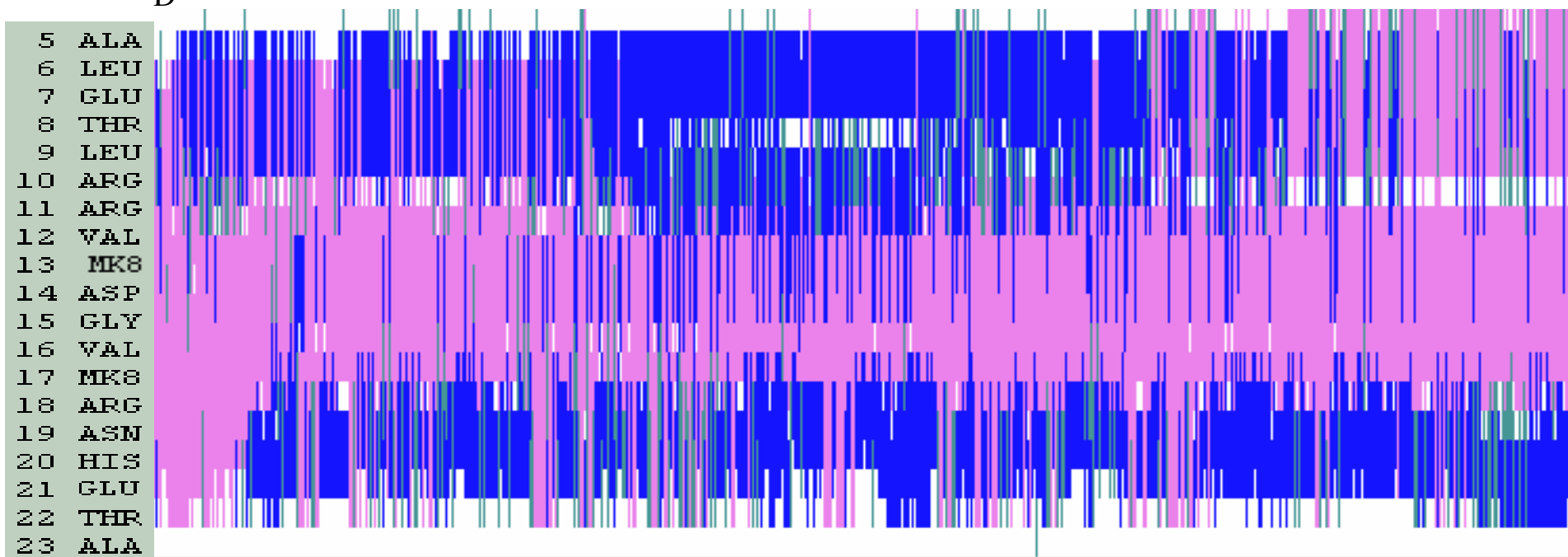

E

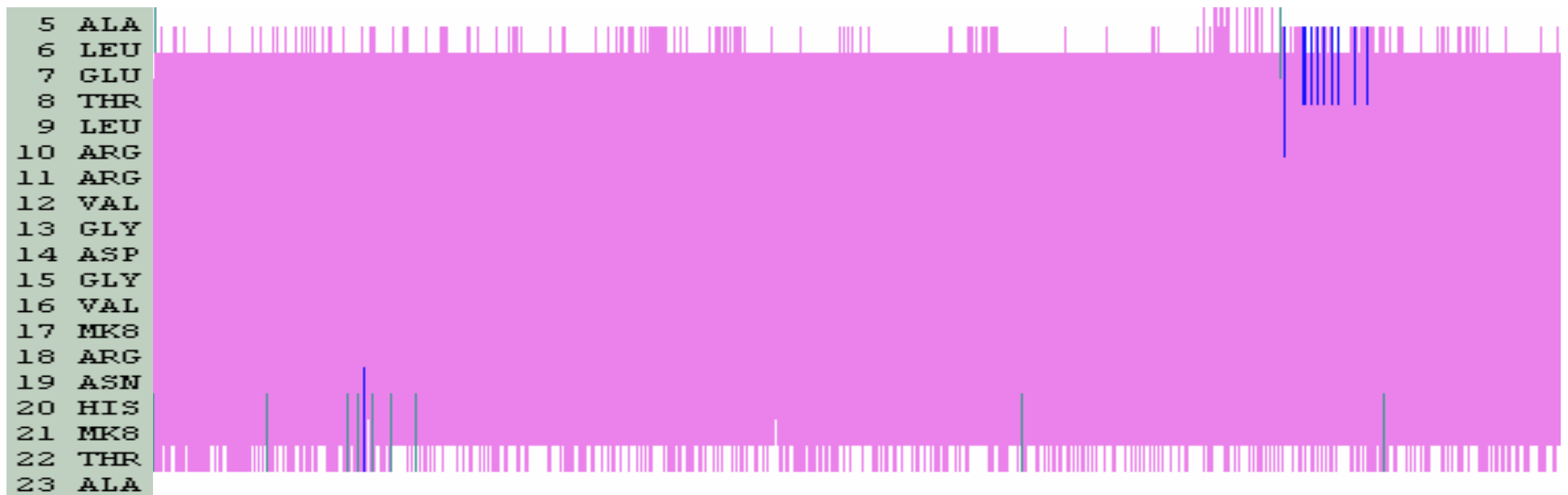

F

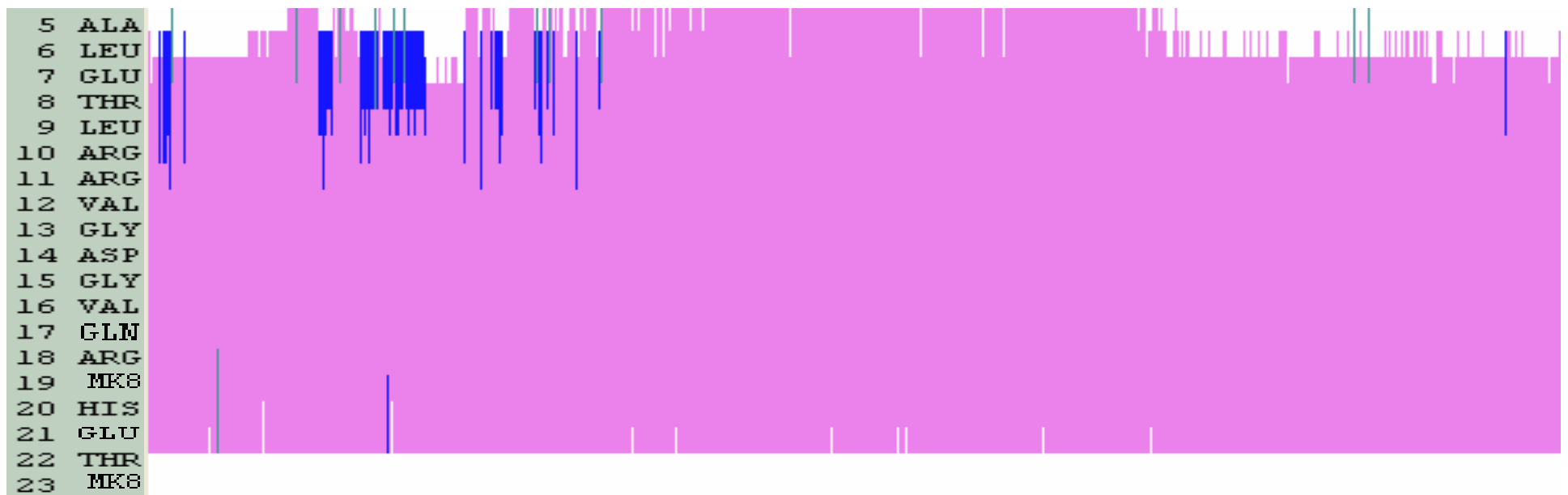

G

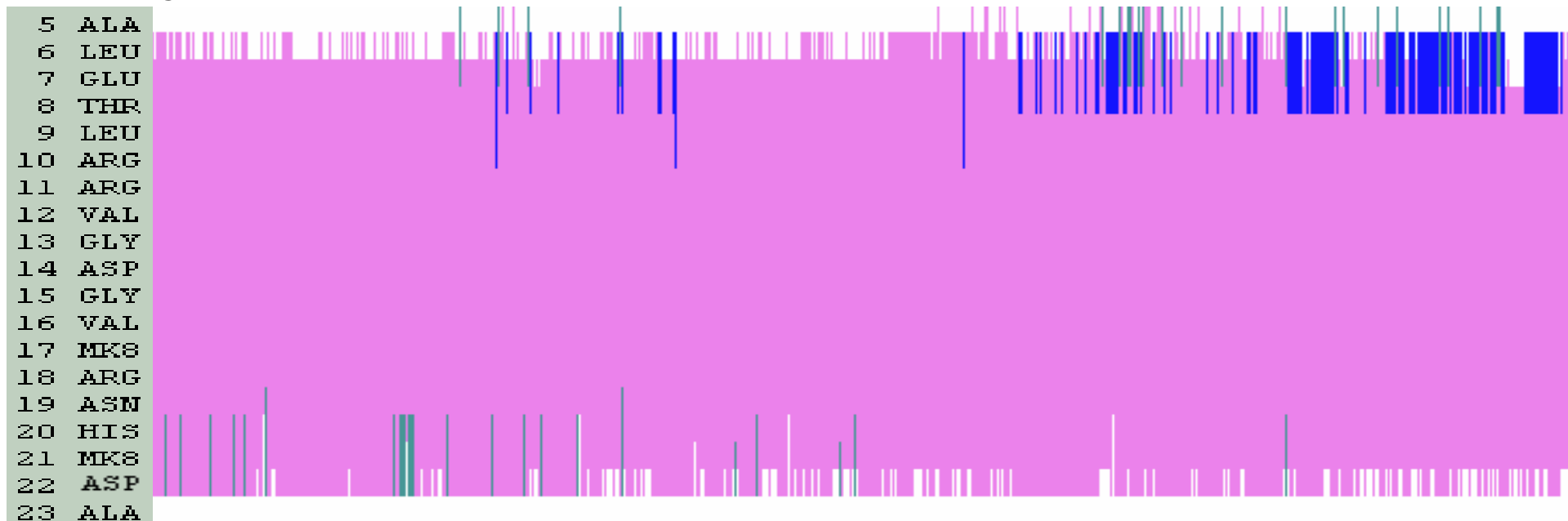

H

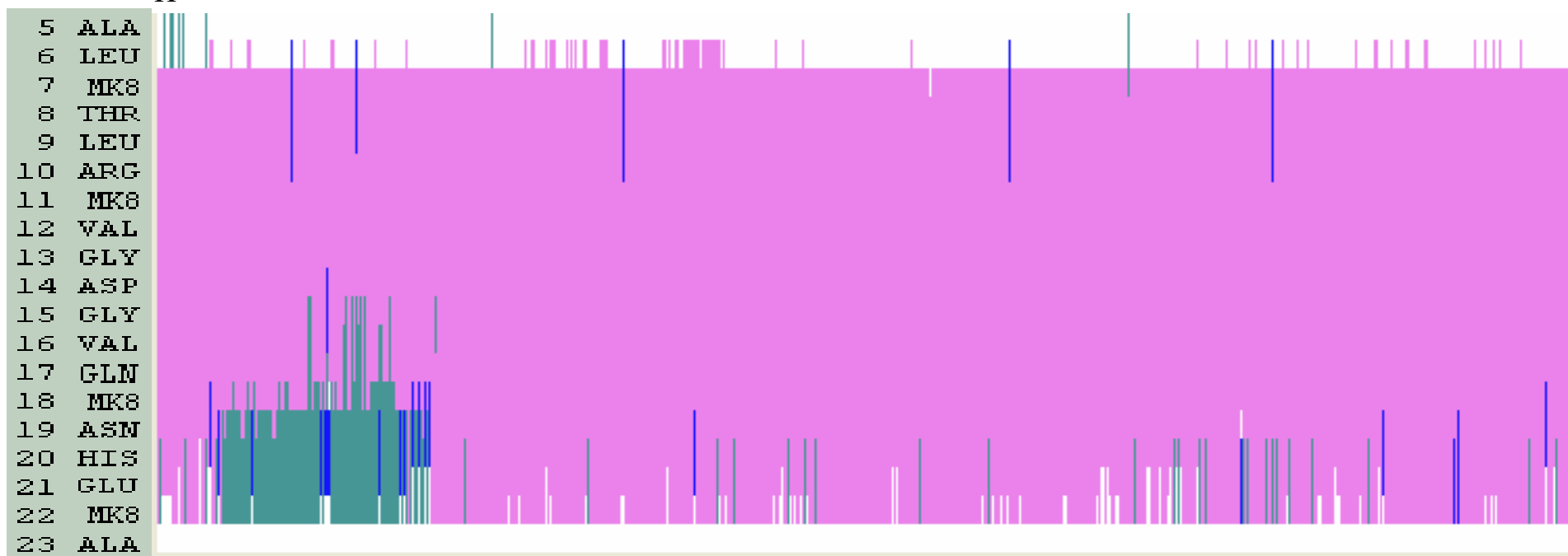

I

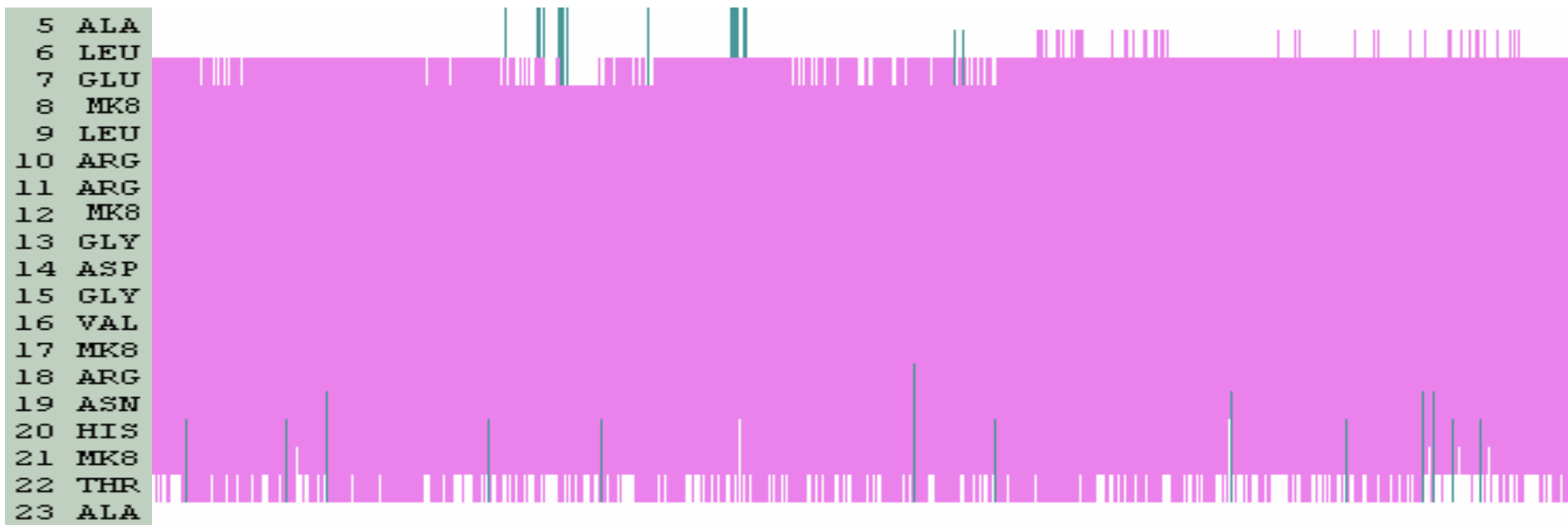

J

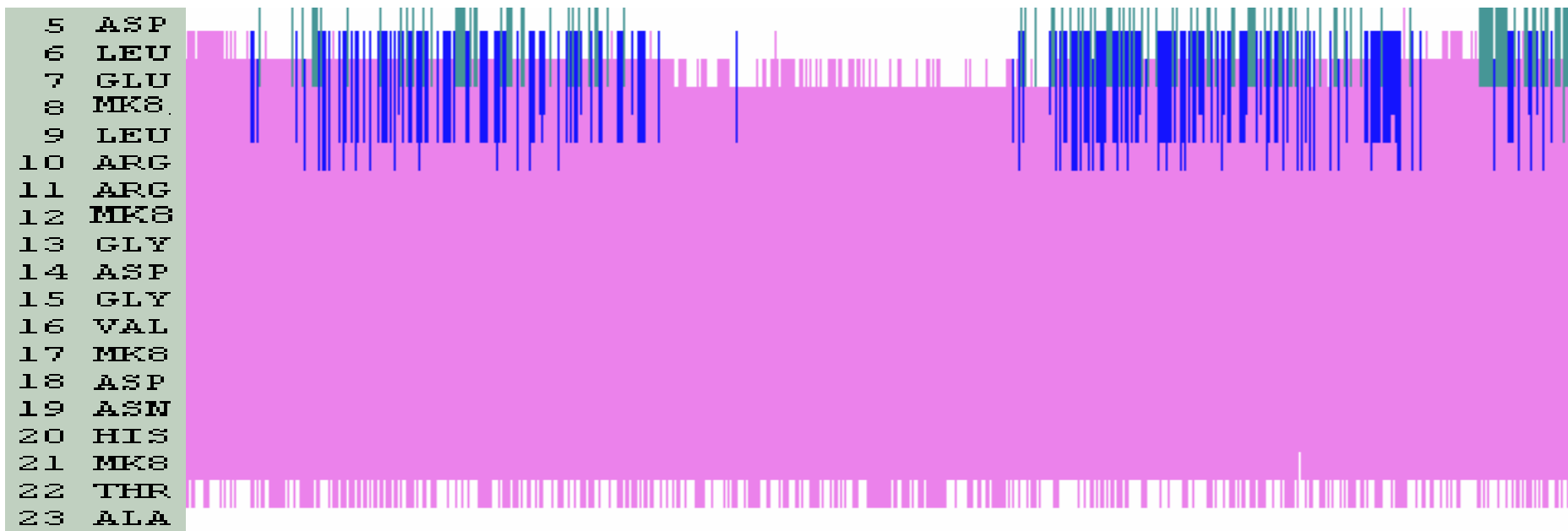

K

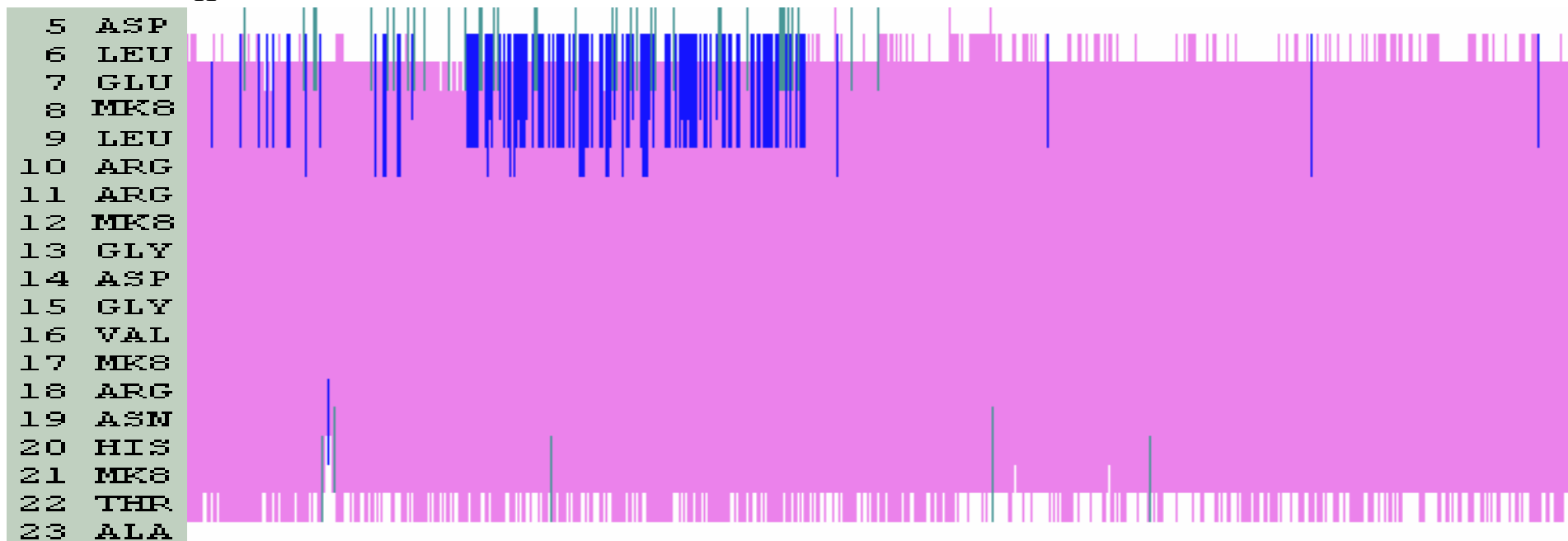

L

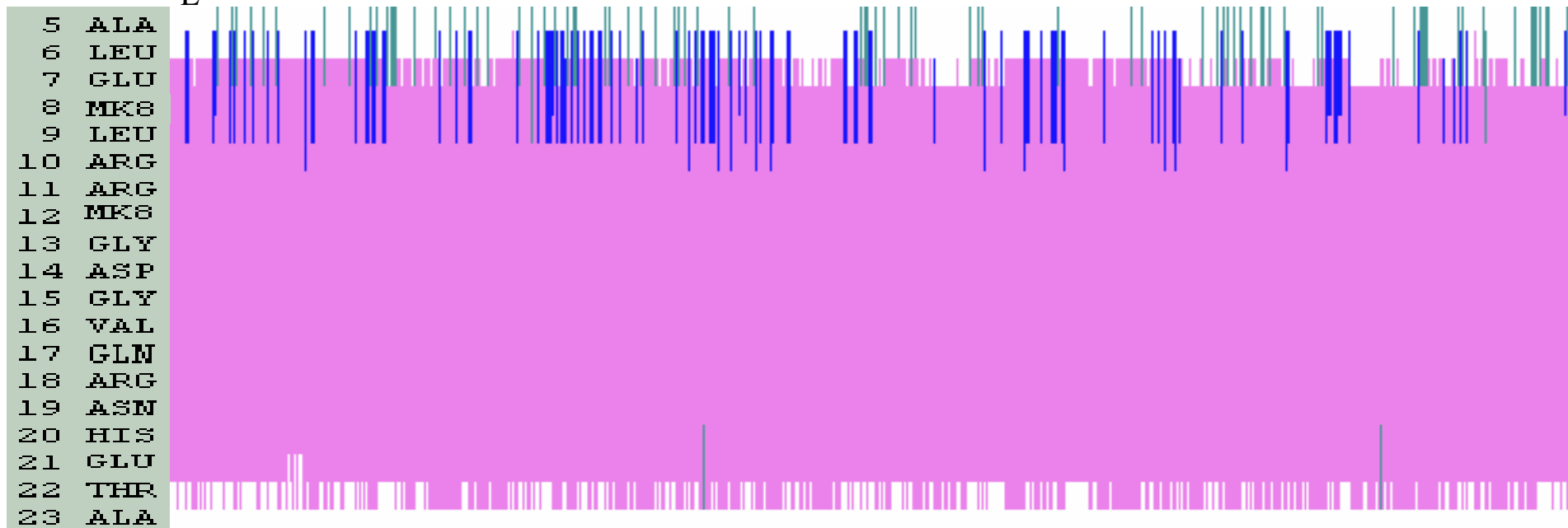

Supplement: Figure S11 — Temporal evolution of the secondary structure profiles of the BH3 peptides when bound with MCL-1 over 20 ns in solution. (A) BH3wt; (B) BH3A; (C) BH3B; (D) BH3C; (E) BH3D; (F) BH3E; (G) BH3F; (H) BH3G; (I) BH3H; (J) BH3I; (K) BH3J and (L) BH3K. When complexed to MCL-1, all peptides except BH3C are helical, especially in the Glu7–Thr22 region. (PDF) [file pone.0043985.s011.pdf]

**Figure S12**

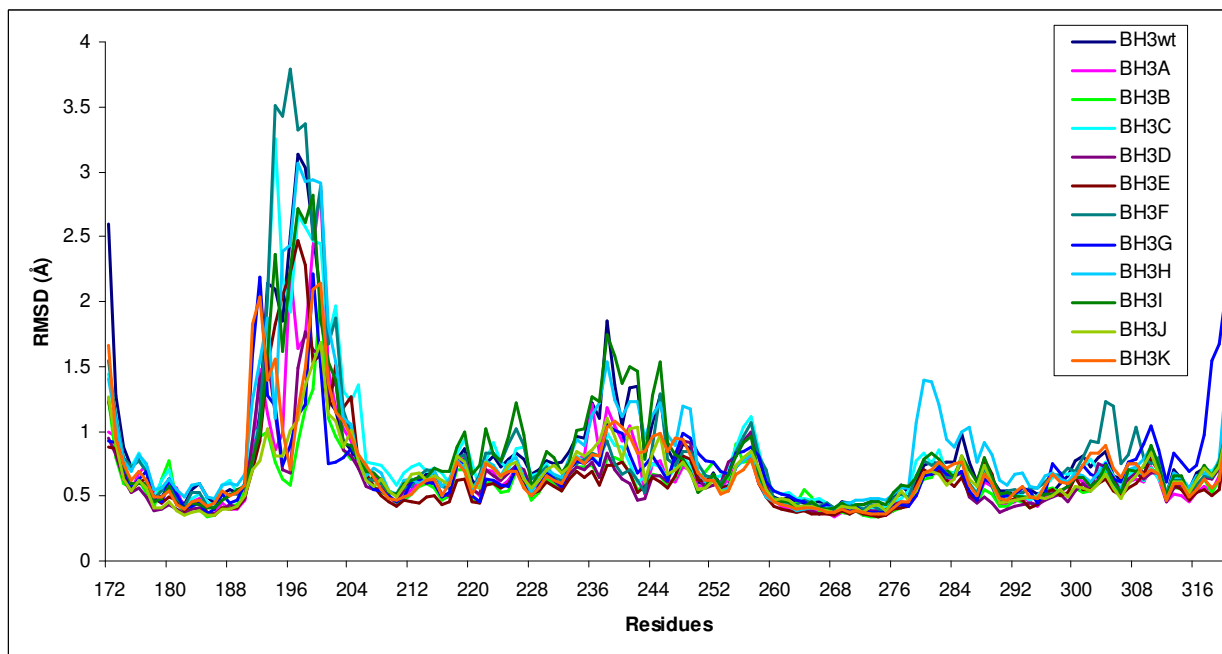

Supplement: Figure S12 — Root mean squared fluctuations for MCL-1 in complex with the BH3 peptides. (PDF) [file pone.0043985.s012.pdf]

**Figure S13**

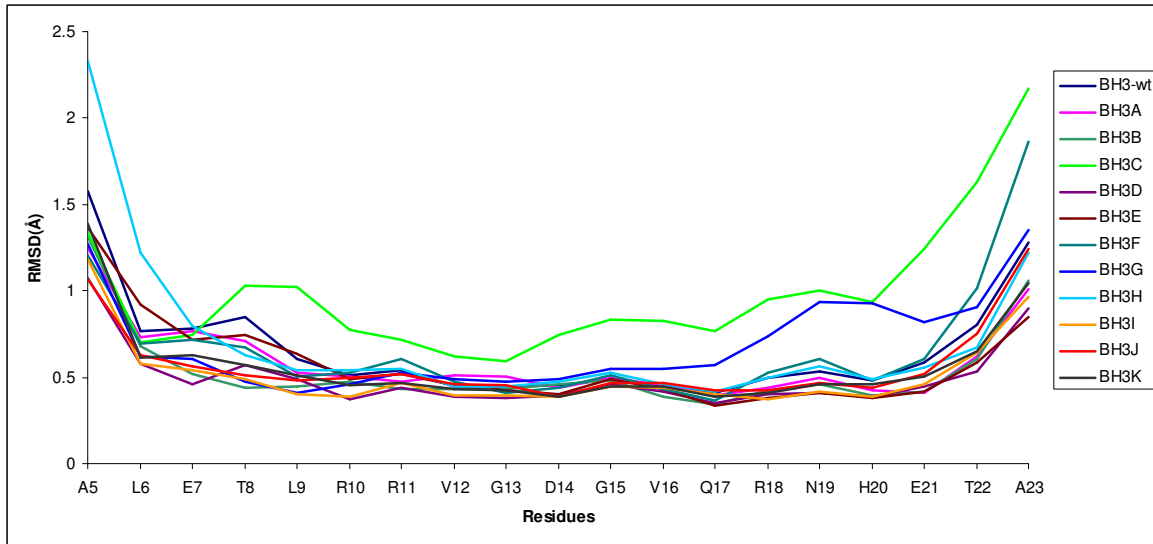

Supplement: Figure S13 — Root mean squared fluctuations for the BH3 peptides in complexes. BH3C peptide show higher fluctuations when compared with all other peptides. (PDF) [file pone.0043985.s013.pdf]

Figure S14

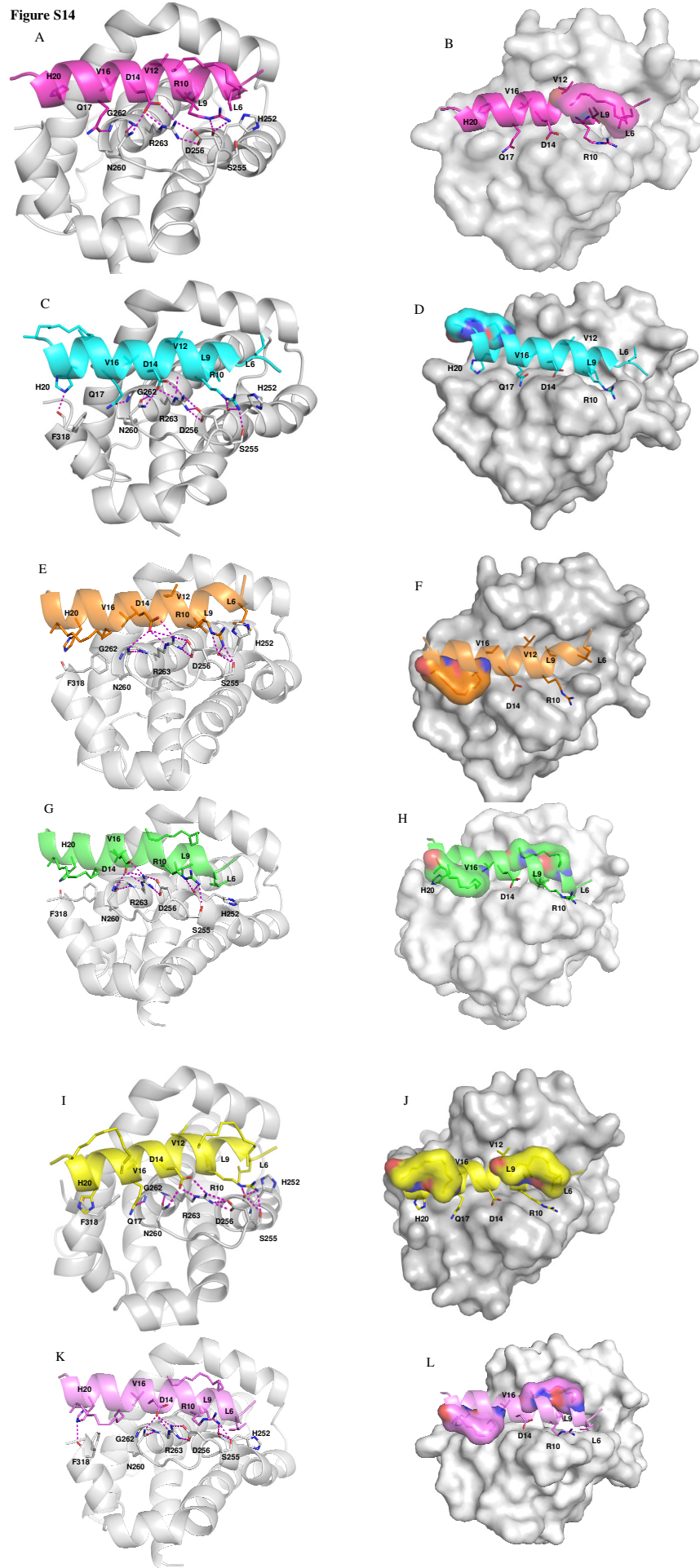

Supplement: Figure S14 — BH3B bound to MCL-1 (shown in grey). (A) Asp14 maintains the hbond network with Arg263, whilst Gln17 makes an hbond interaction with Gly262. (shown in cartoon), (B) The hydrophobic groups Leu6, Leu9, and Val16 are buried in the hydrophobic binding groove on the surface of MCL-1 (shown in surface); BH3E bound to MCL-1 (shown in grey) (C) BH3E staple is less packed against the surface of MCL-1 compared to the staple in the best binder BH3D, but the positioning of the staple enables Gln17 to make hbond interactions with the Gly262 backbone (shown in cartoon), (D) The hydrophobic groups Leu6, Leu9, and Val16 are buried in the hydrophobic binding groove on the surface of MCL-1 (shown in surface); BH3F bound to MCL-1 (shown in grey) (E) Interactions similar to those made by BH3D are also observed in the BH3F peptide bound to MCL-1, with the staple enabling Gln17 to make hbond interactions with the Gly262 backbone (shown in cartoon), (F) The hydrophobic groups Leu6, Leu9, and Val16 are buried in the hydrophobic binding groove on the surface of MCL-1 (shown in surface); BH3G bound to MCL-1 (shown in grey) (G) Double stapling improves the packing of the stapled regions and also maintains the helical content (shown in cartoon), (H) The hydrophobic groups Leu6, Leu9, and Val16 are buried in the hydrophobic binding groove on the surface of MCL-1 (shown in surface); BH3I bound to MCL-1 (shown in grey) (I) Double stapling improves the packing of those stapled regions and also maintains the helical content (shown in cartoon), (J) The hydrophobic groups Leu6, Leu9, and Val16 are buried in the hydrophobic binding groove on the surface of MCL-1 (shown in surface); BHJ bound to MCL-1 (shown in grey) (K) Double stapling improves the packing of the stapled regions and also maintains the helical content (shown in cartoon), (L) The hydrophobic groups Leu6, Leu9, and Val16 are buried in the hydrophobic binding groove on the surface of MCL-1 (shown in surface). (PDF) [file pone.0043985.s014.pdf]
