# Supplementary material for: Stapled BH3 Peptides against MCL-1: Mechanism and Design Using Atomistic Simulations
Source: PLoS One. 2012 Aug 31;7(8):e43985. doi: 10.1371/journal.pone.0043985 (PMC3432064; doi:10.1371/journal.pone.0043985)
Supplement: Table S1 — Binding enthalpies (kcal/mol) of BH3A stapled peptide against MCL-1 using single point computational alanine scanning. (PDF) [file pone.0043985.s015.pdf]

Table S1

	BH3A (R11 and G15 are stapled)	ΔH	Δ
1	BH3A	-78.1	0.0
2	A05E	-74.0	-4.1
3	L06A	-73.4	-4.7
4	E07A	-74.2	-4.0
5	T08A	-77.7	-0.4
6	L09A	-71.5	-6.6
7	R10A	-65.8	-12.3
8	V12A	-76.2	-1.9
9	G13A	-73.7	-4.4
10	D14A	-58.0	-20.1
11	V16A	-74.4	-3.7
12	Q17A	-76.4	-1.7
13	R18A	-78.2	0.0
14	N19A	-78.6	0.4
15	H20A	-74.0	-4.1
16	E21A	-77.3	-0.8
17	T22A	-77.5	-0.7
18	A23E	-77.8	-0.4
