# Supplementary material for: Stapled BH3 Peptides against MCL-1: Mechanism and Design Using Atomistic Simulations
Source: PLoS One. 2012 Aug 31;7(8):e43985. doi: 10.1371/journal.pone.0043985 (PMC3432064; doi:10.1371/journal.pone.0043985)
Supplement: Table S2 — Components of binding free energy (in kcal/mol) of MCL-1 with BH3 peptides. (PDF) [file pone.0043985.s016.pdf]

Table S2

	MCL-1-BH3-wt		MCL-1		BH3-wt		Delta	
	Mean	Std	Mean	Std	Mean	Std	Mean	Std
ELE	-6719.5	70.0	-5649.8	64.6	-941.8	24.6	-127.9	17.1
VDW	-742.3	22.9	-622.3	20.2	-30.6	6.5	-89.3	5.6
GAS	-4586.1	78.0	-3716.0	72.1	-652.7	26.6	-217.3	18.1
GBSOL	-2251.5	62.8	-1928.9	59.0	-472.5	22.1	149.9	16.0
GBTOT	-6837.6	39.2	-5644.9	36.9	-1125.2	12.7	**-67.4**	5.3
TSTOT	1960.4	4.7	1749.3	5.8	251.2	1.6	**-40.1**	5.6
ΔG_{bind}							**-27.3**	

	MCL-1- BH3A		MCL-1		BH3A		Delta	
	Mean	Std	Mean	Std	Mean	Std	Mean	Std
ELE	-6710.5	73.3	-5740.5	64.7	-807.6	51.1	-162.5	29.6
VDW	-763.4	22.0	-640.3	19.3	-28.0	6.5	-95.1	6.1
GAS	-4597.3	78.3	-3838.0	69.0	-501.7	49.4	-257.6	27.3
GBSOL	-2048.7	65.8	-1841.3	55.2	-398.2	45.4	190.8	23.5
GBTOT	-6645.9	39.1	-5679.3	37.3	-899.9	13.1	**-66.8**	6.2
TSTOT	1951.1	5.7	1739.0	6.6	250.9	1.9	**-38.8**	5.4
ΔG_{bind}							**-27.9**	

	MCL-1- BH3B		MCL-1		BH3B		Delta	
	Mean	Std	Mean	Std	Mean	Std	Mean	Std
ELE	-6524.3	78.1	-5771.5	68.1	-672.1	21.9	-80.6	28.3
VDW	-742.3	21.1	-620.4	19.5	-30.3	6.0	-91.6	4.9
GAS	-4393.4	82.5	-3841.7	73.2	-379.5	24.5	-172.2	28.2
GBSOL	-2185.7	69.6	-1834.5	60.2	-460.8	19.7	109.7	22.4
GBTOT	-6579.0	37.7	-5676.2	35.7	-840.3	12.1	**-62.5**	8.3
TSTOT	1952.4	5.8	1744.0	5.4	245.3	1.8	**-36.9**	5.3
ΔG_{bind}							**-25.6**	

	MCL-1- BH3C		MCL-1		BH3C		Delta	
	Mean	Std	Mean	Std	Mean	Std	Mean	Std
ELE	-6676.7	72.4	-5754.1	67.4	-887.3	26.0	-35.4	20.7
VDW	-729.4	20.9	-619.3	19.5	-18.6	7.0	-91.4	5.7
GAS	-4523.4	77.9	-3817.7	72.7	-578.9	27.3	-126.8	22.7
GBSOL	-2203.6	63.3	-1844.8	58.1	-432.7	22.9	73.9	20.1
GBTOT	-6726.9	40.1	-5662.5	37.6	-1011.5	13.0	**-52.9**	5.2
TSTOT	1962.3	6.6	1748.8	5.6	255.1	1.9	**-41.6**	6.3
ΔG_{bind}							**-11.2**	

	MCL-1- BH3D		MCL-1		BH3D		Delta	
	Mean	Std	Mean	Std	Mean	Std	Mean	Std
ELE	-6631.5	75.9	-5768.8	73.2	-818.2	14.2	-44.5	15.0
VDW	-755.5	20.5	-637.7	19.3	-32.2	6.3	-85.6	5.1
GAS	-4515.3	82.9	-3858.6	80.3	-526.6	16.6	-130.1	15.7
GBSOL	-2178.5	65.1	-1814.9	62.6	-430.5	11.4	66.9	14.0
GBTOT	-6693.8	39.5	-5673.5	37.7	-957.1	11.8	**-63.2**	4.7
TSTOT	1956.3	5.7	1744.3	4.5	248.9	1.3	**-36.9**	4.6
ΔG_{bind}							**-26.3**	

	MCL-1- BH3E		MCL-1		BH3E		Delta	
	Mean	Std	Mean	Std	Mean	Std	Mean	Std
ELE	-6716.6	89.9	-5725.3	78.5	-931.5	42.8	-59.8	14.3
VDW	-755.7	20.0	-629.6	18.4	-32.5	6.7	-93.6	4.5
GAS	-4583.3	92.5	-3804.9	83.0	-625.0	43.3	-153.4	14.9
GBSOL	-2099.8	76.7	-1867.5	68.7	-317.5	39.1	85.3	13.4
GBTOT	-6683.1	39.3	-5672.4	37.4	-942.5	12.9	**-68.1**	4.6
TSTOT	1956.5	4.9	1745.4	5.8	252.4	1.7	**-41.3**	4.4
ΔG_{bind}							**-26.8**	

	MCL-1- BH3F		MCL-1		BH3F		Delta	
	Mean	Std	Mean	Std	Mean	Std	Mean	Std
ELE	-6572.1	67.9	-5688.1	62.0	-796.0	26.5	-88.0	15.6
VDW	-750.2	20.9	-632.9	20.0	-29.9	5.8	-87.4	5.2
GAS	-4450.7	75.0	-3776.7	68.1	-498.7	27.7	-175.3	16.7
GBSOL	-2269.4	59.8	-1890.4	53.4	-489.8	25.1	110.8	14.5
GBTOT	-6720.1	38.3	-5667.1	36.2	-988.5	12.4	**-64.6**	4.9
TSTOT	1957.2	5.6	1744.3	5.8	251.3	1.4	**-38.4**	5.4
ΔG_{bind}							**-26.1**	

	MCL-1- BH3G		MCL-1		BH3G		Delta	
	Mean	Std	Mean	Std	Mean	Std	Mean	Std
ELE	-6410.2	75.7	-5831.2	67.3	-393.0	13.0	-186.0	22.9
VDW	-749.7	20.2	-630.5	19.3	-27.7	5.9	-91.5	5.0
GAS	-4292.5	82.5	-3919.0	73.5	-96.0	17.6	-277.5	24.1
GBSOL	-2054.8	66.3	-1768.1	58.5	-496.0	11.0	209.3	21.7
GBTOT	-6347.3	38.2	-5687.2	36.2	-592.0	12.4	**-68.2**	5.4
TSTOT	1945.3	3.8	1742.3	4.5	244.7	1.1	**-41.7**	4.2
ΔG_{bind}							**-26.4**	

	MCL-1- BH3H		MCL-1		BH3H		Delta	
	Mean	Std	Mean	Std	Mean	Std	Mean	Std
ELE	-6447.0	75.4	-5651.0	71.5	-758.3	25.1	-37.7	19.4
VDW	-739.8	22.0	-624.0	20.2	-29.2	6.3	-86.6	4.4
GAS	-4295.9	80.6	-3725.5	74.4	-446.1	28.7	-124.4	19.4
GBSOL	-2292.9	63.3	-1933.7	62.8	-414.9	23.3	55.7	17.9
GBTOT	-6588.8	41.4	-5659.2	37.8	-860.9	13.3	**-68.7**	4.7
TSTOT	1962.2	5.5	1743.5	5.5	254.1	3.1	**-35.4**	5.1
ΔG_{bind}							**-33.4**	

	MCL-1- BH3I		MCL-1		BH3I		Delta	
	Mean	Std	Mean	Std	Mean	Std	Mean	Std
ELE	-6711.9	73.9	-5736.1	63.2	-851.1	19.7	-124.6	32.4
VDW	-754.9	22.7	-632.1	20.8	-30.7	6.6	-92.1	5.3
GAS	-4584.2	85.0	-3828.8	72.8	-538.8	21.1	-216.7	32.6
GBSOL	-2092.4	68.6	-1847.1	56.2	-390.3	16.9	145.0	30.6
GBTOT	-6676.6	38.8	-5675.9	37.2	-929.1	12.4	**-71.7**	5.9
TSTOT	1964.0	5.4	1743.9	5.2	258.4	2.1	**-38.3**	5.6
ΔG_{bind}							**-33.5**	

	MCL-1- BH3J		MCL-1		BH3J		Delta	
	Mean	Std	Mean	Std	Mean	Std	Mean	Std
ELE	-6569.6	72.1	-5688.1	67.7	-637.0	15.8	-244.5	26.4
VDW	-749.8	20.5	-629.2	19.2	-30.0	6.1	-90.7	5.1
GAS	-4438.9	78.0	-3767.6	74.6	-336.2	18.8	-335.1	25.3
GBSOL	-2120.4	62.9	-1904.9	57.9	-480.4	13.4	264.9	24.1
GBTOT	-6559.3	38.9	-5672.5	36.7	-816.6	13.0	**-70.3**	4.5
TSTOT	1956.1	5.1	1744.2	5.2	250.5	1.6	**-38.5**	4.5
ΔG_{bind}							**-31.7**	

	MCL-1- BH3K		MCL-1		BH3K		Delta	
	Mean	Std	Mean	Std	Mean	Std	Mean	Std
ELE	-6768.9	80.8	-5780.1	69.5	-858.2	35.1	-130.6	18.9
VDW	-750.6	21.9	-625.0	19.9	-32.2	6.5	-93.5	5.2
GAS	-4643.2	83.4	-3862.1	71.4	-557.0	36.8	-224.1	20.0
GBSOL	-2143.1	68.5	-1815.7	58.0	-478.7	31.9	151.4	18.3
GBTOT	-6786.3	38.4	-5677.8	35.4	-1035.7	12.7	**-72.7**	4.7
TSTOT	1959.1	6.1	1743.6	5.7	254.6	2.7	**-39.0**	5.3
ΔG_{bind}							**-33.7**	
