# Supplementary material for: Stapled BH3 Peptides against MCL-1: Mechanism and Design Using Atomistic Simulations
Source: PLoS One. 2012 Aug 31;7(8):e43985. doi: 10.1371/journal.pone.0043985 (PMC3432064; doi:10.1371/journal.pone.0043985)
Supplement: Table S3 — Residuewise energy contributions (in kcal/mol) of BH3 peptides for its interactions with MCL-1. (PDF) [file pone.0043985.s017.pdf]

Table S3

Residues	BH3-wt	BH3A	BH3B	BH3C	BH3D	BH3E	BH3F	BH3G	BH3H	BH3I	BH3J	BH3K
A5	5.3	6.5	6.6	4.5	5.1	5.7	4.8	6.7	3.7	6.3	5.0	5.9
L6	-5.5	-5.5	-5.7	-5.5	-4.7	-5.3	-5.1	-5.3	-3.8	-5.9	-5.3	-5.5
E7	-0.7	-1.6	0.0	-1.2	-0.7	-1.0	-0.8	0.0	-0.7	-1.3	-1.3	-0.8
T8	-0.4	0.2	-0.7	0.3	-0.6	-0.9	-0.2	-0.6	0.0	0.0	0.0	0.0
L9	-4.9	-5.0	-5.1	-4.0	-4.8	-5.0	-4.5	-4.8	-4.5	-4.5	-4.5	-4.5
R10	-5.4	-5.3	-4.3	-3.6	-5.3	-4.5	-5.3	-5.1	-5.9	-5.2	-4.6	-5.8
R11	0.6	0.0	-2.9	0.5	0.6	0.7	0.3	-3.1	0.4	0.5	0.5	0.5
V12	-3.2	-3.0	-2.9	-4.6	-3.1	-3.0	-2.8	-3.2	-7.3	-6.3	-7.0	-7.4
G13	-1.3	-1.6	-1.6	0.0	-1.0	-1.6	-1.1	-1.6	-1.5	-1.4	-1.4	-1.7
D14	-3.1	-3.4	-2.9	-0.1	-2.8	-3.2	-3.0	-3.2	-3.0	-3.3	-3.2	-3.2
G15	0.1	-1.9	0.1	0.0	0.2	0.2	0.2	0.2	0.2	0.2	0.2	0.2
V16	-2.9	-3.1	-3.2	-3.9	-2.8	-3.2	-3.1	-2.7	-3.0	-2.9	-3.0	-2.9
Q17	-1.7	-2.2	-2.2	-6.0	0.0	-2.3	0.0	-1.9	0.0	0.0	0.0	-2.0
R18	0.5	0.8	0.3	0.5	0.3	0.3	0.4	0.0	0.4	0.4	0.1	0.3
N19	0.6	0.5	0.5	-0.4	0.5	0.0	0.5	0.4	0.5	0.5	0.5	0.5
H20	-1.6	-0.8	-1.2	-1.3	-1.8	-1.3	-2.2	-1.6	-1.3	-1.9	-2.0	-2.0
E21	0.4	0.1	0.3	0.4	-4.3	0.1	-4.6	0.5	-4.5	-4.2	-4.3	0.3
T22	0.1	0.1	0.1	0.1	0.1	0.2	0.2	-0.4	0.1	0.1	0.1	0.1
A23	0.4	0.5	0.5	0.3	0.3	-1.0	0.4	0.1	0.4	0.4	0.4	0.4
