# Supplementary material for: Stapled BH3 Peptides against MCL-1: Mechanism and Design Using Atomistic Simulations
Source: PLoS One. 2012 Aug 31;7(8):e43985. doi: 10.1371/journal.pone.0043985 (PMC3432064; doi:10.1371/journal.pone.0043985)
Supplement: Table S4 — Binding enthalpies (kcal/mol) of BH3-wt peptide against MCL-1 using single point computational alanine scanning. (PDF) [file pone.0043985.s018.pdf]

Table S4

	**BH3-wt**	**ΔH**	**Δ**
1	wt	-75.0	0.0
2	A05E	-71.7	-3.4
3	L06A	-71.3	-3.8
4	E07A	-71.4	-3.6
5	T08A	-73.7	-1.3
6	L09A	-69.4	-5.7
7	R10A	-63.8	-11.2
8	R11A	-76.6	1.6
9	V12A	-72.9	-2.1
10	G13A	-72.1	-2.9
11	D14A	-55.7	-19.3
12	G15A	-76.0	1.0
13	V16A	-71.9	-3.2
14	Q17A	-73.9	-1.1
15	R18A	-76.7	1.7
16	N19A	-75.5	0.4
17	H20A	-70.9	-4.1
18	E21A	-75.4	0.3
19	T22A	-75.5	0.4
20	A23E	-75.5	0.5
